# Overcoming the curse of dimensionality in the numerical approximation of high-dimensional semilinear elliptic partial differential equations

**DOI:** 10.1007/s42985-024-00272-4

**Published:** 2024-10-11

**Authors:** Christian Beck, Lukas Gonon, Arnulf Jentzen

**Affiliations:** 1 Department of Mathematics, ETH Zurich, Zurich, Switzerland; 2 Department of Mathematics, Imperial College London, London, UK; 3 School of Data Science and Shenzhen Research Institute of Big Data, The Chinese University of Hong Kong (CUHK-Shenzhen), Shenzhen, China; 4 Applied Mathematics, Institute for Analysis and Numerics, Faculty of Mathematics and Computer Science, University of Münster, Münster, Germany

**Keywords:** Numerical analysis, Monte Carlo methods, Full-history recursive multilevel Picard approximations, Semilinear elliptic partial differential equations, 65Cxx, 65C05, 65Nxx, 65N75

## Abstract

Recently, so-called full-history recursive multilevel Picard (MLP) approximation schemes have been introduced and shown to overcome the curse of dimensionality in the numerical approximation of semilinear *parabolic* partial differential equations (PDEs) with Lipschitz nonlinearities. The key contribution of this article is to introduce and analyze a new variant of MLP approximation schemes for certain semilinear *elliptic* PDEs with Lipschitz nonlinearities and to prove that the proposed approximation schemes overcome the curse of dimensionality in the numerical approximation of such semilinear elliptic PDEs.

## Introduction

Partial differential equations (PDEs) are widely used as a modelling tool, e.g., in the natural sciences, in the engineering sciences, or in the financial industry. Usually the exact solutions to specific PDE problems in applications can hardly be found. Thus, there is a high demand for approximative solution techniques. In the scientific literature, there are several well-established approximation methods for PDEs like finite difference methods or finite element methods which work well in low dimensions. However, such classical deterministic approximation methods cannot be used in high dimensions as they suffer from the so-called *curse of dimensionality* in the sense that the computational effort for calculating an approximation grows at least exponentially in the PDE dimension $d \in \mathbb {N}$. Probabilistic approximation methods like Monte Carlo averaging, on the other hand, do not suffer from the curse of dimensionality in the numerical approximation of linear second-order parabolic PDEs as well as linear second-order elliptic PDEs.

Recently, several probabilistic approximation methods for high-dimensional nonlinear PDEs have been proposed in hopes of overcoming the curse of dimensionality in the numerical approximation of nonlinear PDEs. We refer, e.g., to [3–5, 9, 10, 15, 17, 21, 22, 25, 27, 32, 37–39, 41, 44, 53–55, 58, 61] for deep learning based approximation methods for possibly nonlinear PDEs, e.g., to [1, 11, 13, 14, 16, 40, 42, 43, 56, 59, 62, 63] for approximation methods for nonlinear second-order parabolic PDEs based on branching diffusions, and, e.g., to [8, 23, 24, 28, 45, 47–50] for full-history recursive multilevel Picard (MLP) approximation methods for nonlinear second-order parabolic PDEs. Numerical experiments raise hopes that deep learning based approximation methods are able to approximate solutions of high-dimensional nonlinear PDE problems, but at the moment there are only partial explanations for the good performance of deep learning based approximation methods in numerical experiments for high-dimensional PDEs available (cf., e.g., [12, 26, 29–31, 33–35, 46, 51, 52, 60]). In contrast to this, there are, however, complete mathematical analyses in the scientific literature showing that branching diffusion approximation methods can efficiently solve high-dimensional semilinear parabolic PDE problems for sufficiently small time horizons. The branching diffusion approximation method, however, breaks down when the time horizon is not sufficiently small anymore. MLP approximation methods are, to the best of our knowledge, up to now the only approximation methods for high-dimensional semilinear parabolic PDEs which are guaranteed by rigorous mathematical proofs to overcome the curse of dimensionality for all time horizons (see [8, 28, 47, 48]). The MLP approximation schemes proposed and studied so far in the scientific literature, however, exclusively deal with parabolic PDE problems and none of these schemes and their analyses can be applied to nonlinear elliptic PDEs. The key contribution of this article is to introduce and analyze MLP approximation schemes for certain semilinear elliptic PDEs and to prove for the first time that approximations of such semilinear elliptic PDEs can be obtained without suffering from the curse of dimensionality. In particular, the main result of this article, Theorem 3.16, proves that the computational effort to obtain an approximation of a desired accuracy $\varepsilon \in (0,\infty )$ grows at most polynomially in the PDE dimension $d\in \mathbb {N}$ as well as in the reciprocal of the desired accuracy. To illustrate the findings of this article in more detail, we present in the following result, Theorem 1.1 below, a special case of Theorem 3.16.

### Theorem 1.1

Let $c,L \in [0,\infty )$, $\lambda \in (L,\infty )$, $M \in \mathbb {N}\cap ( (\sqrt{\lambda }+\sqrt{L})^2(\sqrt{\lambda }-\sqrt{L})^{-2}, \infty )$, $\Theta = \cup _{n\in \mathbb {N}} \mathbb {Z}^n$, let $f_d \in C(\mathbb {R}^d\times \mathbb {R},\mathbb {R})$, $d \in \mathbb {N}$, let $( \Omega , \mathcal {F}, \mathbb {P})$ be a probability space, let $W^{d,\theta } :[0,\infty ) \times \Omega \rightarrow \mathbb {R}^d$, $\theta \in \Theta$, $d \in \mathbb {N}$, be i.i.d. standard Brownian motions, let $R^{\theta } :\Omega \rightarrow [0,\infty )$, $\theta \in \Theta$, be i.i.d. random variables, assume that $(R^{\theta })_{\theta \in \Theta }$ and $(W^{d,\theta })_{(d,\theta )\in \mathbb {N}\times \Theta }$ are independent, assume for all $d \in \mathbb {N}$, $x=(x_1,\ldots ,x_d) \in \mathbb {R}^d$, $v,w \in \mathbb {R}$, $\varepsilon \in (0,\infty )$ that

1 $$\begin{aligned} \textstyle | f_d(x,v) - f_d(x,w) - \lambda ( v - w ) | \leqslant L | v-w |, \quad | f_d(x,0) | \leqslant c d^c \bigl [ 1 + \sum _{ j = 1 }^d | x_j | \bigr ]^c, \end{aligned}$$

and $\mathbb {P}(R^0 \geqslant \varepsilon ) = e^{-\lambda \varepsilon }$, let $U^{d,\theta }_{n} = (U^{d,\theta }_{n}(x))_{x\in \mathbb {R}^d}:\mathbb {R}^d\times \Omega \rightarrow \mathbb {R}$, $\theta \in \Theta$, $d,n\in \mathbb {N}_0$, satisfy for all $d,n \in \mathbb {N}$, $\theta \in \Theta$, $x \in \mathbb {R}^d$ that $U^{d,\theta }_{0}(x) = 0$ and

2 $$\begin{aligned} \begin{aligned}&U^{d,\theta }_{n}(x) = \frac{-1}{\lambda M^n} \left[ \sum _{m=1}^{M^n} f_d\big (x+\sqrt{2}\,W^{d,(\theta ,0,m)}_{R^{(\theta ,0,m)}},0\big ) \right] \\&+ \sum _{k=1}^{n-1} \frac{1}{\lambda M^{n-k}} \left[ \sum _{m=1}^ {M^{n-k}} \bigg ( \lambda \left[ U^{d,(\theta ,k,m)}_{k}\big (x+\sqrt{2}\,W^{d,(\theta ,k,m)}_{R^{(\theta ,k,m)}}\big )\right. \right. \\&-\left. U^{d,(\theta ,k,-m)}_{k-1}\big (x+\sqrt{2}\,W^{d,(\theta ,k,m)}_{R^{(\theta ,k,m)}}\big ) \right] \\&- \left[ f_d\!\left( x+\sqrt{2}\,W^{d,(\theta ,k,m)}_{R^{(\theta ,k,m)}}, U^{d,(\theta ,k,m)}_{k}\big (x+\sqrt{2}\,W^{d,(\theta ,k,m)}_{R^{(\theta ,k,m)}}\big ) \right) \right. \\&\left. \left. - f_d\!\left( x+\sqrt{2}\,W^{d,(\theta ,k,m)}_{R^{(\theta ,k,m)}}, U^{d,(\theta ,k,-m)}_{k-1}\big (x+\sqrt{2}\,W^{d,(\theta ,k,m)}_{R^{(\theta ,k,m)}}\big ) \right) \right] \bigg ) \right] , \end{aligned} \end{aligned}$$

and let $\mathfrak {C}_{d,n} \in \mathbb {R}$, $d,n\in \mathbb {N}_0$, satisfy for all $d,n \in \mathbb {N}_0$ that $\mathfrak {C}_{d,n} \leqslant (d+1) M^n + \sum _{k=1}^{n-1} M^{n-k} ( d + 1 + \mathfrak {C}_{d,k} + \mathfrak {C}_{d,k-1} )$. Then

(i) there exist unique $u_d \in C(\mathbb {R}^d,\mathbb {R})$, $d \in \mathbb {N}$, with $\forall \, d \in \mathbb {N}$, $\varepsilon \in (0,\infty ) :\sup _{ x=( x_1, \dots , x_d ) \in \mathbb {R}^d } [ | u_d(x) | \exp ( - \varepsilon \sum _{ j = 1 }^d | x_j | ) ] < \infty$ which satisfy for all $d \in \mathbb {N}$ that $u_d$ is a viscosity solution of
  3 $$\begin{aligned} (\Delta u_d)(x) = f_d(x,u_d(x)) \end{aligned}$$
  for $$ x \in \mathbb {R}^d $$ and
(ii) there exist $\kappa \in \mathbb {R}$ and $\mathfrak {N} :(0,1] \times \mathbb {N}\rightarrow \mathbb {N}$ such that for all $d \in \mathbb {N}$, $\varepsilon \in (0,1]$ it holds that 4

Theorem 1.1 is an immediate consequence of Corollary 3.17 and Corollary 2.10. Corollary 3.17, in turn, follows from Theorem 3.16, the main result of this article. In the following we add comments on some of the mathematical objects appearing in Theorem 1.1. The functions $u_d :\mathbb {R}^d \rightarrow \mathbb {R}$, $d \in \mathbb {N}$, in Theorem 1.1 above describe the solutions of the elliptic PDEs which we intend to solve approximately; see (3) above. The functions $f_d :\mathbb {R}^d \times \mathbb {R}\rightarrow \mathbb {R}$, $d \in \mathbb {N}$, represent the nonlinearities in the elliptic PDEs in (3). The nonlinearities are assumed to satisfy certain Lipschitz conditions which are formulated with the help of the real numbers $L \in [0,\infty )$ and $\lambda \in (L,\infty )$ in Theorem 1.1 above.

The random fields $U^{d,\theta }_n :\mathbb {R}^d \times \Omega \rightarrow \mathbb {R}$, $d \in \mathbb {N}$, $n \in \mathbb {N}_0$, $\theta \in \Theta$, in (2) above describe the approximation algorithm which we employ in this work to approximately calculate the PDE solutions $u_d :\mathbb {R}^d \rightarrow \mathbb {R}$, $d \in \mathbb {N}$. The set $\Theta = \cup _{n\in \mathbb {N}} \mathbb {Z}^n$ is a sufficiently large index set to appropriately describe independence properties of random variables. For example, observe that for all $n \in \mathbb {N}$, $\theta =(\theta _1,\ldots ,\theta _n) \in \mathbb {Z}^n \subseteq \Theta$, $k \in \mathbb {N}$, $m \in \mathbb {N}$ it holds that $(\theta ,k,m)=(\theta _1,\theta _2,\ldots ,\theta _n,k,m) \in \mathbb {Z}^{n+2} \subseteq \Theta$ and $(\theta ,k,-m)=(\theta _1,\theta _2,\ldots ,\theta _n,k,-m) \in \mathbb {Z}^{n+2} \subseteq \Theta$. Combining this and (2) with the assumption that $(R^{\theta })_{\theta \in \Theta }$ are independent and the fact that for all $d \in \mathbb {N}$ it holds that $(W^{d,\theta })_{\theta \in \Theta }$ are independent ensures that for all $d,m,k \in \mathbb {N}$, $\theta \in \Theta$, $x \in \mathbb {R}^d$ it holds that

5 $$\begin{aligned} U^{d,(\theta ,k,m)}_k(x) \qquad \text {and} \qquad U^{d,(\theta ,k,-m)}_{k-1}(x) \end{aligned}$$

are independent.

The motivation for the specific form of the random fields $U^{d,\theta }_n :\mathbb {R}^d \times \Omega \rightarrow \mathbb {R}$, $d \in \mathbb {N}$, $n \in \mathbb {N}_0$, $\theta \in \Theta$, in (2) stems from multilevel Monte Carlo approximations of Picard approximations of certain stochastic fixed point equations (SFPEs) which are satisfied by the solutions $u_d :\mathbb {R}^d \rightarrow \mathbb {R}$, $d \in \mathbb {N}$, of the elliptic PDEs in (3). The different numbers of Monte Carlo samples in (2) are determined by the constant $M \in \mathbb {N}$ which is restricted by the real numbers $L \in [0,\infty )$ and $\lambda \in (L,\infty )$ used to formulate the Lipschitz assumptions for the nonlinearities $f_d :\mathbb {R}^d \times \mathbb {R}\rightarrow \mathbb {R}$, $d \in \mathbb {N}$, in the PDEs in (3) above. For every $d,n \in \mathbb {N}$, $x \in \mathbb {R}^d$ we think of the quantity $\mathfrak {C}_{d,n} \in \mathbb {R}$ in (4) in Theorem 1.1 as the computational cost to sample one realization of $U^{d,0}_n(x) \in \mathbb {R}$ (cf. Sect. 3.6 below). Theorem 1.1 thus, roughly speaking, proves that the random fields $U^{d,0}_n :\mathbb {R}^d \times \Omega \rightarrow \mathbb {R}$, $d \in \mathbb {N}$, $n \in \mathbb {N}_0$, (see (2) in Theorem 1.1) can achieve an approximation accuracy of size $\varepsilon \in (0,\infty )$ with a computational cost which is bounded by a polynomial in the PDE dimension $d \in \mathbb {N}$ and in the reciprocal of the desired approximation accuracy $\varepsilon \in (0,\infty )$ (see (4) in Theorem 1.1). The real number $c \in [0,\infty )$ is an arbitrarily large real constant which we use to express the growth assumption in Theorem 1.1 that for all $d \in \mathbb {N}$, $x=(x_1,\ldots ,x_d) \in \mathbb {R}^d$ it holds that $|f_d(x,0)| \leqslant c d^c [1+\sum _{j=1}^d |x_j|]^c$ as well as to specify the regions $[-c,c]^d \subseteq \mathbb {R}^d$, $d \in \mathbb {N}$, on which we measure the $L^2$-error between the exact solutions $u_d :\mathbb {R}^d \rightarrow \mathbb {R}$, $d \in \mathbb {N}$, of the PDEs in (3) and the random approximations $U^{d,0}_{\mathfrak {N}_{\varepsilon ,d}} :\mathbb {R}^d \times \Omega \rightarrow \mathbb {R}$, $d \in \mathbb {N}$, $\varepsilon \in (0,1]$, in (4) in Theorem 1.1 above.

Theorem 1.1 ensures that there exist real numbers $\kappa _0, \kappa _1,\kappa _2 \in \mathbb {R}$ and $\mathfrak {N} :(0,1] \times \mathbb {N}\rightarrow \mathbb {N}$ such that for all $d \in \mathbb {N}$, $\varepsilon \in (0,1]$ it holds that6but it does not provide any information on the size of the constants $\kappa _0, \kappa _1,\kappa _2$ and on the way how these constants depend on the real numbers $c,L \in [0,\infty )$, $\lambda \in (L,\infty )$, $M \in \mathbb {N}\cap ( (\sqrt{\lambda }+\sqrt{L})^2(\sqrt{\lambda }-\sqrt{L})^{-2}, \infty )$ used to formulate the assumptions of Theorem 1.1. However, our refined statement Theorem 3.16 in Sect. 3.7 below gives a precise estimate on the constants $\kappa _1$, $\kappa _2$ in dependence of the real numbers used to formulate the assumptions of Theorem 3.16. To develop a better understanding of the assumptions and conclusions of Theorem 1.1 above we now illustrate Theorem 1.1 by means of a simple example.

### Example 1.2

Let $\lambda = 4$ and for every $d\in \mathbb {N}$ let $f_d :\mathbb {R}^d \times \mathbb {R}\rightarrow \mathbb {R}$ satisfy for all $d \in \mathbb {N}$, $x \in \mathbb {R}^d$, $u \in \mathbb {R}$ that $f_d(x,u)= 4 u + \sin (u+x)$. Then the PDEs in (7) read as

7 $$\begin{aligned} (\Delta u_d)(x) = 4 u_d(x) + \sin (x+u_d(x)) \end{aligned}$$

for $x \in \mathbb {R}^d$, $d \in \mathbb {N}$. Next observe that it holds for all $d \in \mathbb {N}$, $x \in \mathbb {R}^d$, $v,w \in \mathbb {R}$ that

8 $$\begin{aligned} | f_d(x,v) - f_d(x,w) - \lambda ( v - w ) | \leqslant | v - w| \qquad \text {and} \qquad | f_d(x,0) | \leqslant 1. \end{aligned}$$

Theorem 1.1 can thus be applied to (7). Moreover, we observe that the more general statement in Theorem 3.16 in Sect. 3.7 below can also be applied to (7) to obtain that for all $M \in \mathbb {N}\cap (9, \infty )$ there exist $\kappa \in \mathbb {R}$ and $\mathfrak {N} :(0,1] \times \mathbb {N}\rightarrow \mathbb {N}$ such that for all $\varepsilon \in (0,1]$, $d \in \mathbb {N}$ it holds that  and9

We now further motivate the specific form of the MLP approximation scheme in (2) in Theorem 1.1. Our approach to derive the MLP approximation schemes for elliptic PDEs in (2) is based on the MLP approximation schemes for parabolic PDEs and related problems in [47]. More specifically, the key idea is to first use the fact that solutions to certain semilinear elliptic PDEs can be identified with solutions of certain SFPEs and then use MLP approximation schemes to approximately calculate the solutions to these SFPEs. Let $d \in \mathbb {N}$ and assume, for the sake of an example, that $u \in C^2(\mathbb {R}^d, \mathbb {R})$, $\lambda \in (0, \infty )$, $g \in C(\mathbb {R}^d, \mathbb {R})$ satisfy for all $x \in \mathbb {R}^d$ that $\lambda u(x) - (\Delta u)(x) = g(x, u(x))$. Under suitable assumptions the function *u* satisfies for all $x \in \mathbb {R}^d$ the SFPE

10 $$\begin{aligned} u(x) = \mathbb {E}\! \left[ \int _0^{\infty } e^{-\lambda t} g(x + \sqrt{2} W_t, u(x+ \sqrt{2} W_t)) \,dt \right] , \end{aligned}$$

where $W :[0,\infty ) \times \Omega \rightarrow \mathbb {R}^d$ is a standard Brownian motion, see Proposition 2.7 below. Employing an ${\text {Exp}}(\lambda )$-distributed random variable $R :\Omega \rightarrow (0, \infty )$ this can be reformulated as

11 $$\begin{aligned} u(x) = \frac{1}{\lambda } \mathbb {E}\! \left[ g( x+ \sqrt{2} W_R, u( x+ \sqrt{2}W_R ) ) \right] . \end{aligned}$$

To this SFPE we apply the well-known Picard iteration method, that is, we recursively introduce functions $U_n :\mathbb {R}^d \rightarrow \mathbb {R}$, $n \in \mathbb {N}$, which satisfy for all $n \in \mathbb {N}$, $x \in \mathbb {R}^d$ that $U_0(x) = 0$ and

12 $$\begin{aligned} \begin{aligned} U_n(x)&= \frac{1}{\lambda } \mathbb {E}\! \left[ g( x + \sqrt{2} W_R, U_{n-1}(x + \sqrt{2} W_R) ) \right] . \end{aligned} \end{aligned}$$

In the next step a telescopic sum argument ensures that

13 $$\begin{aligned}&U_n(x) = \frac{1}{\lambda } \mathbb {E}\! \left[ g( x + \sqrt{2} W_R, 0) \right] \nonumber \\&+ \sum _{l=1}^{n-1} \frac{1}{\lambda } \mathbb {E}\! \left[ g( x + \sqrt{2} W_R, U_{l}(x + \sqrt{2} W_R) ) - g( x + \sqrt{2} W_R, U_{l-1} (x + \sqrt{2} W_R) ) \right] . \end{aligned}$$

Thus, we have expressed $U_n$ in terms of expectations of $U_0,\ldots ,U_{n-1}$ and we now apply a multilevel Monte Carlo approach to approximate these expectations by sums with a carefully chosen number of Monte Carlo samples for each summand. In the situation where $f_d :\mathbb {R}^d \times \mathbb {R}\rightarrow \mathbb {R}$ in Theorem 1.1 satisfies for all $x \in \mathbb {R}^d, v \in \mathbb {R}$ that $f_d(x,v) = \lambda v - g(x, v)$, this leads precisely to the MLP approximation scheme in (2).

The remainder of this article is organized as follows. Section 2 is devoted to the study of stochastic representations for suitable viscosity solutions of certain semilinear elliptic PDEs. In particular, we establish in Sect. 2 a one-to-one correspondence between suitable viscosity solutions of certain semilinear elliptic PDEs and solutions of SFPEs associated with such semilinear elliptic PDEs. Section 3 focusses on the introduction and the analysis of MLP schemes for the numerical approximation of solutions of certain SFPEs. Owing to the results in Sect. 2 these MLP schemes are thus apt to numerically approximate suitable viscosity solutions of certain semilinear elliptic PDEs. The main error estimates for the MLP approximation schemes can be found in Sects. 3.4 and 3.5 and a computational cost analysis for the MLP approximation schemes is carried out in Sect. 3.6. An overall complexity analysis for the MLP approximation schemes is obtained in Sect. 3.7 by combining the error estimates from Sects. 3.4 and 3.5 with the computational cost analysis in Sect. 3.6.

## Stochastic representations for elliptic partial differential equations (PDEs)

In the main result of this section, Proposition 2.7 in Sect. 2.3 below, we establish a stochastic representation formula of the Feynman–Kac type for suitable viscosity solutions of certain semilinear elliptic PDEs (cf. also Corollary 2.8 and Corollary 2.10 in Sect. 2.3). The established Feynman–Kac type formula will be essential in our error analysis for MLP approximations in Sect. 3 below. Our proof of Proposition 2.7 is particularly based on the following three tools: (i) an existence and uniqueness result for SFPEs, see Corollary 2.2 in Sect. 2.1 below, (ii) a result which identifies solutions of certain SFPEs as viscosity solutions of certain semilinear elliptic PDEs, see Corollary 2.5 in Sect. 2.1, and (iii) a uniqueness result for suitable viscosity solutions of certain semilinear elliptic PDEs, see Proposition 2.6 in Sect. 2.2 below. Our proof of the existence and uniqueness result in Corollary 2.2 in Sect. 2.1 (see (i) above) is based on Proposition 2.1, which can be deduced by an elementary application of Banach’s fixed point theorem (cf. also [6]). The identification result for certain semilinear elliptic PDEs in Corollary 2.5 in Sect. 2.1 (see (ii) above) follows from an approximation argument which combines a well-known convergence result for viscosity solutions (cf., e.g., [36, Lemma 4.8] or [2, Theorem A.2]) with the well-known construction result for classical PDE solutions in Lemma 2.3 below (cf., e.g., [20, Theorems 7.4.5 and 7.5.1]). Our proof of the uniqueness result for suitable viscosity solutions of certain semilinear elliptic PDEs in Proposition 2.6 in Sect. 2.2 (see (iii) above) is inspired by [18, Theorem 2.1].

### On stochastic fixed point equations (SFPEs) and semilinear elliptic PDEs

In this subsection we prove existence and uniqueness of solutions to SFPEs under a Lyapunov-type condition, see Proposition 2.1 below. We then specialize this result to prove an existence and uniqueness result for SFPEs related to Brownian motion, see Corollary 2.2 below. Finally, in Corollary 2.5 below, we prove a result which identifies solutions of such SFPEs as viscosity solutions of certain semilinear elliptic PDEs by combining classical Feynman–Kac type representations with stability properties of viscosity solutions (cf. Lemma 2.3 and Lemma 2.4).

We start with an elementary existence and uniqueness result for SFPEs in Proposition 2.1 below. A similar result for SFPEs associated to parabolic PDEs can be found in [6, Theorem 2.9]. Our proof of Proposition 2.1 consists of an application of Banach’s fixed point theorem.

#### Proposition 2.1

Let $d \in \mathbb {N}$, $L, \rho \in \mathbb {R}$, $\lambda \in (L+\rho ,\infty )$, let $\left\| \cdot \right\| :\mathbb {R}^d \rightarrow [0,\infty )$ be a norm on $\mathbb {R}^d$, let $\mathcal {O} \subseteq \mathbb {R}^d$ be a non-empty open set, for every $r \in (0,\infty )$ let $O_r \subseteq \mathcal {O}$ satisfy , let $( \Omega , \mathcal {F}, \mathbb {P} )$ be a probability space, for every $x \in \mathcal {O}$ let $X^x = (X^x_t)_{t\in [0,\infty )}:[0,\infty ) \times \Omega \rightarrow \mathcal {O}$ be measurable, assume for all $\varepsilon ,t \in (0,\infty )$ and all $x_n \in \mathcal {O}$, $n \in \mathbb {N}_0$, with $\limsup _{n\rightarrow \infty } \left\| x_n-x_0 \right\| = 0$ that $\limsup _{n\rightarrow \infty } \mathbb {P}( \left\| X^{x_n}_t-X^{x_0}_t \right\| \geqslant \varepsilon ) = 0$, let $V \in C( \mathcal {O}, (0,\infty ) )$, $f \in C( \mathcal {O} \times \mathbb {R}, \mathbb {R})$ satisfy for all $t\in [0,\infty )$, $x \in \mathcal {O}$, $v,w \in \mathbb {R}$ that $\mathbb {E}[ e^{-\rho t} V(X^x_t) ] \leqslant V(x)$ and $|f(x,v) - f(x,w)| \leqslant L|v-w|$, and assume that $\inf _{r\in (0,\infty )} [\sup _{x\in \mathcal {O}{{\setminus }} O_r} (\frac{|f(x,0)|}{V(x)})] = 0$ and $\sup _{r \in (0,\infty ) } [\inf _{x\in \mathcal {O}{{\setminus }} O_r} V(x)] = \infty$. Then there exists a unique $u \in C(\mathcal {O},\mathbb {R})$ such that it holds for all $x \in \mathcal {O}$ that $\limsup _{r\rightarrow \infty } [ \sup _{y\in \mathcal {O} {{\setminus }} O_r} ( \frac{|u(y)|}{V(y)} ) ] = 0$, $\mathbb {E}\! \left[ \int _0^{\infty } e^{-\lambda t} |f(X^x_t,u(X^x_t))|\,dt \right] < \infty$, and

14 $$\begin{aligned} u(x) = \mathbb {E}\! \left[ \int _0^{\infty } e^{-\lambda t} f(X^x_t,u(X^x_t))\,dt \right] \!. \end{aligned}$$

#### Proof of Proposition 2.1

For the sake of brevity, we only sketch a proof of Proposition 2.1 and refer to Section 2.1 (in particular to Proposition 2.6 therein) of this article’s preprint version [7] for a complete proof of Proposition 2.1. Let $\mathcal {V}= \{ u \in C(\mathcal {O},\mathbb {R}):\limsup _{r\rightarrow \infty } [ \sup _{x\in \mathcal {O}{{\setminus }} O_r} (\frac{|u(x)|}{V(x)}) ] = 0 \}$ and let $\left\| \cdot \right\| _{\mathcal {V}}:\mathcal {V}\rightarrow [0,\infty )$ satisfy for all $v \in \mathcal {V}$ that $\left\| v \right\| _{\mathcal {V}} = \sup _{x\in \mathcal {O}} ( \frac{|v(x)|}{V(x)} )$. Note that $(\mathcal {V}, \left\| \cdot \right\| _{\mathcal {V}})$ is an $\mathbb {R}$-Banach space. Moreover, observe that there exists a unique function $\Phi :\mathcal {V}\rightarrow \mathcal {V}$ which satisfies for all $x \in \mathcal {O}$, $v \in \mathcal {V}$ that $[\Phi (v)](x) = \mathbb {E}\! \left[ \int _0^{\infty } e^{-\lambda t} f(X^x_t,v(X^x_t))\,dt \right]$. Furthermore, it holds for all $v,w\in \mathcal {V}$ that $\left\| \Phi (v)-\Phi (w) \right\| _{\mathcal {V}} \leqslant \frac{L}{\lambda - \rho } \left\| v-w \right\| _{\mathcal {V}}$. This, the hypothesis that $\lambda >L+\rho$, the fact that $(\mathcal {V}, \left\| \cdot \right\| _{\mathcal {V}})$ is an $\mathbb {R}$-Banach space, and Banach’s fixed point theorem demonstrate that there exists a unique $u\in \mathcal {V}$ which satisfies $\Phi (u)=u$. This completes the sketch of our proof of Proposition 2.1. $\square$

In the next result, we specialize Proposition 2.1 above to the situation in which $\mathcal {O}= \mathbb {R}^d$ and $X^x$ is a linearly transformed Brownian motion started in $x \in \mathbb {R}^d$. Thereby, we obtain an existence and uniqueness result for SFPEs related to Brownian motion. As we will see later on, solutions to these SFPEs are in fact solutions to certain semilinear Poisson PDEs. By applying Proposition 2.1 to solutions of more general Itô stochastic differential equations we could also obtain existence and uniqueness results for SFPEs related to more general semilinear elliptic PDEs, but for the sake of conciseness, we do not pursue this direction further in the present article.

#### Corollary 2.2

Let $d,m\in \mathbb {N}$, $B \in \mathbb {R}^{d\times m}$, $L, \rho \in \mathbb {R}$, $\lambda \in (\rho +L,\infty )$, let $\left\| \cdot \right\| :\mathbb {R}^d\rightarrow [0,\infty )$ be a norm on $\mathbb {R}^d$, let $f\in C(\mathbb {R}^d\times \mathbb {R},\mathbb {R})$, $V\in C^2(\mathbb {R}^d,(0,\infty ))$ satisfy for all $x\in \mathbb {R}^d$, $v,w\in \mathbb {R}$ that $|f(x,v)-f(x,w)|\leqslant L|v-w|$ and

15 $$\begin{aligned} \tfrac{1}{2}{\text {Trace}}( BB^{*}({\text {Hess}} V)(x) ) \leqslant \rho V(x), \end{aligned}$$

assume that $\limsup _{r\rightarrow \infty } [\sup _{ \left\| x \right\| >r} (\frac{|f(x,0)|}{V(x)})] = 0$ and $\sup _{r\in (0,\infty )} [\inf _{ \left\| x \right\| >r} V(x)] = \infty$, let $(\Omega ,\mathcal {F},\mathbb {P})$ be a probability space, and let $W:[0,\infty )\times \Omega \rightarrow \mathbb {R}^m$ be a standard Brownian motion. Then there exists a unique $u\in C(\mathbb {R}^d,\mathbb {R})$ such that for all $x \in \mathbb {R}^d$ it holds that

16 $$\begin{aligned}  &   \textstyle \limsup _{r\rightarrow \infty } [\sup _{ \left\| y \right\| >r} (\frac{|u(y)|}{V(y)})] = 0, \end{aligned}$$

17 $$\begin{aligned}  &   \quad \textstyle \mathbb {E}\! \left[ \int _0^{\infty } e^{-\lambda s} |f(x+BW_s,u(x+BW_s))|\,ds \right] < \infty , \end{aligned}$$

18 $$\begin{aligned}  &   \text {and} \qquad \textstyle {u(x) = \mathbb {E}\! \left[ \int _0^{\infty } e^{-\lambda s} f(x+BW_s,u(x+BW_s))\,ds \right] }. \end{aligned}$$

#### Proof of Corollary 2.2

First, note that combining [6, Lemmas 3.1 and 3.2] with (15) ensures that for all $t\in [0,\infty )$, $x \in \mathbb {R}^d$ it holds that

19 $$\begin{aligned} \mathbb {E}\! \left[ e^{-\rho t}V(x+BW_t) \right] \leqslant V(x). \end{aligned}$$

Furthemore, observe that for all $\varepsilon , t \in (0,\infty )$ and all $x_n \in \mathbb {R}^d$, $n \in \mathbb {N}_0$, with $\limsup _{ n \rightarrow \infty } \left\| x_n - x_0 \right\| = 0$ it holds that

20 $$\begin{aligned} \limsup _{ n \rightarrow \infty } \mathbb {P}( \left\| (x_n+BW_t)-(x_0+BW_t) \right\| \geqslant \varepsilon ) = 0. \end{aligned}$$

Combining Proposition 2.1 and (19) hence demonstates that there exists a unique $u \in C(\mathbb {R}^d,\mathbb {R})$ such that for all $x \in \mathbb {R}^d$ it holds that $\limsup _{ r \rightarrow \infty } [ \sup _{ \left\| y \right\| >r} ( \frac{|u(y)|}{V(y)} ) ] = 0$, $\mathbb {E}\big [ \int _0^{\infty } e^{-\lambda s} |f(x+BW_s,u(x+BW_s))|\,ds \big ] < \infty$, and

21 $$\begin{aligned} \textstyle {u(x) = \mathbb {E}\! \left[ \int _0^{\infty } e^{-\lambda t} f(x+BW_t,u(x+BW_t))\,dt \right] }. \end{aligned}$$

This completes the proof of Corollary 2.2. $\square$

The remaining results of this subsection are dedicated to establishing that in the setting of Corollary 2.2 above the function *u* is a viscosity solution of a certain semilinear elliptic PDE. In Lemma 2.3 below we state a well-known Feynman–Kac type representation for the related parabolic problem.

#### Lemma 2.3

Let $T \in (0,\infty )$, $d,m \in \mathbb {N}$, $B \in \mathbb {R}^{d\times m}$, $\varphi \in C^{2}(\mathbb {R}^d,\mathbb {R})$ satisfy $\sup _{x\in \mathbb {R}^d} [ \sum _{i,j=1}^d ( | \varphi (x) | + | (\frac{ \partial }{\partial x_i}\varphi )(x) | + |(\frac{\partial ^2}{\partial x_i\partial x_j} \varphi )(x)| ) ] < \infty$, let $(\Omega ,\mathcal {F},\mathbb {P})$ be a probability space, let $W :[0,T] \times \Omega \rightarrow \mathbb {R}^m$ be a standard Brownian motion, and let $u :[0,T] \times \mathbb {R}^d \rightarrow \mathbb {R}$ satisfy for all $t \in [0,T]$, $x \in \mathbb {R}^d$ that $u(t,x) = \mathbb {E}\! \left[ \varphi (x+BW_t) \right]$. Then

(i) it holds that $u \in C^{1,2}([0,T] \times \mathbb {R}^d, \mathbb {R})$ and
(ii) it holds for all $t \in [0,T]$, $x \in \mathbb {R}^d$ that
  22 $$\begin{aligned} (\tfrac{\partial u}{\partial t})(t,x) = \tfrac{1}{2} {\text {Trace}}\!\big (BB^{*}({\text {Hess}}_x u)(t,x)\big ). \end{aligned}$$

#### Proof of Lemma 2.3

We refer to the literature for a proof of this classical result. A detailed proof can also be found, e.g., in the extended preprint version [7, Lemma 2.8] of this article. $\square$

In Lemma 2.4 below we establish a Feynman–Kac type representation result for viscosity solutions of certain inhomogeneous linear elliptic PDEs. Our proof of this result proceeds, first, by inferring the desired Feynman–Kac type representation for classical solutions from the representation in Lemma 2.3 and, thereafter, by an appropriate limiting argument. Lemma 2.4 is similar to related Feynman–Kac type representation results in the scientific literature such as [57, Theorem 2.4]. Lemma 2.4 slightly differs from [57, Theorem 2.4] in the sense that the Lyapunov function $V \in C^2( \mathbb {R}^d, (0,\infty ) )$ appears in the statement of Lemma 2.4.

#### Lemma 2.4

Let $d,m \in \mathbb {N}$, $B \in \mathbb {R}^{d\times m}$, $\lambda \in (0,\infty )$, $\rho \in (-\infty ,\lambda )$, let $\left\| \cdot \right\| :\mathbb {R}^d \rightarrow [0,\infty )$ be a norm on $\mathbb {R}^d$, let $h \in C( \mathbb {R}^d, \mathbb {R})$, $V \in C^2( \mathbb {R}^d, (0,\infty ) )$, assume for all $x \in \mathbb {R}^d$ that

23 $$\begin{aligned} \tfrac{1}{2}{\text {Trace}}(BB^{*}({\text {Hess}} V)(x)) \leqslant \rho V(x), \end{aligned}$$

assume that $\sup _{ r \in (0,\infty ) } [ \inf _{ \left\| x \right\| >r} V(x) ] = \infty$ and $\inf _{ r \in (0,\infty ) } [ \sup _{ \left\| x \right\| > r } ( \frac{| h(x) |}{ V(x) } ) ] = 0$, let $( \Omega , \mathcal {F}, \mathbb {P})$ be a probability space, let $W :[0,\infty ) \times \Omega \rightarrow \mathbb {R}^m$ be a standard Brownian motion, and let $u :\mathbb {R}^d \rightarrow \mathbb {R}$ satisfy for all $x \in \mathbb {R}^d$ that

24 $$\begin{aligned} u(x) = \mathbb {E}\! \left[ \int _0^{\infty } e^{-\lambda s} h(x+BW_s) \,ds \right] \end{aligned}$$

(cf. Corollary 2.2). Then it holds that *u* is a viscosity solution of

25 $$\begin{aligned} \lambda u(x) - \tfrac{1}{2} {\text {Trace}}(BB^{*}({\text {Hess}} u)(x)) = h(x) \end{aligned}$$

for $$ x \in \mathbb {R}^d $$.

#### Proof of Lemma 2.4

Throughout this proof let $\mathfrak {h}_n \in C^{\infty }(\mathbb {R}^d,\mathbb {R})$, $n \in \mathbb {N}$, be compactly supported functions which satisfy

26 $$\begin{aligned} \limsup _{n \rightarrow \infty } \left[ \sup _{x\in \mathbb {R}^d} \left( \frac{| \mathfrak {h}_n(x) - h(x) |}{V(x)} \right) \right] = 0, \end{aligned}$$

let $F_n :\mathbb {R}^d \times \mathbb {R}\times \mathbb {R}^d \times \mathbb {S}_d \rightarrow \mathbb {R}$, $n \in \mathbb {N}_0$, satisfy for all $n \in \mathbb {N}$, $x,p \in \mathbb {R}^d$, $r \in \mathbb {R}$, $A \in \mathbb {S}_d$ that

27 $$\begin{aligned} F_n(x,r,p,A) = \lambda r - \tfrac{1}{2} {\text {Trace}}(BB^{*}A) - \mathfrak {h}_n(x) \end{aligned}$$

and

28 $$\begin{aligned} F_0(x,r,p,A) = \lambda r - \tfrac{1}{2} {\text {Trace}}(BB^{*}A) - h(x), \end{aligned}$$

let $\mathfrak {u}_n :\mathbb {R}^d \rightarrow \mathbb {R}$, $n \in \mathbb {N}$, satisfy for all $n \in \mathbb {N}$, $x \in \mathbb {R}^d$ that

29 $$\begin{aligned} \mathfrak {u}_n(x) = \mathbb {E}\! \left[ \int _0^{\infty } e^{-\lambda s} \, \mathfrak {h}_n(x+BW_s)\,ds \right] \!, \end{aligned}$$

and let $\mathfrak {v}_n :[0,\infty ) \times \mathbb {R}^d \rightarrow \mathbb {R}$, $n \in \mathbb {N}$, satisfy for all $n \in \mathbb {N}$, $t \in [0,\infty )$, $x \in \mathbb {R}^d$ that $\mathfrak {v}_n(t,x) = \mathbb {E}[ \mathfrak {h}_n(x+BW_t) ]$. Observe that Lemma 2.3 ensures for all $n \in \mathbb {N}$, $t \in [0,\infty )$, $x \in \mathbb {R}^d$ that $\mathfrak {v}_n \in C^{1,2}([0,\infty )\times \mathbb {R}^d,\mathbb {R})$ and $(\tfrac{\partial }{\partial t}\mathfrak {v}_n)(t,x) = \tfrac{1}{2} {\text {Trace}}(BB^{*}({\text {Hess}}_x \mathfrak {v}_n)(t,x) )$. This, (29), the fact that for all $n \in \mathbb {N}$ it holds that $\sup _{(t,x)\in [0,\infty ) \times \mathbb {R}^d} [ \sum _{i,j=1}^d (|\mathfrak {v}_n(t,x)|+|(\tfrac{\partial }{\partial x_i}\mathfrak {v}_n)(t,x)|+|(\tfrac{\partial ^2}{\partial x_i\partial x_j}\mathfrak {v}_n)(t,x)|)] < \infty$, integration by parts, and Lebesgue’s dominated convergence theorem guarantee that for all $n \in \mathbb {N}$, $x \in \mathbb {R}^d$ it holds that

30 $$\begin{aligned} \begin{aligned} \mathfrak {u}_n(x)&= \int _0^{\infty } e^{-\lambda t} \, \mathfrak {v}_n(t,x) \,dt = \lim _{R\rightarrow \infty } \left[ \int _0^R e^{-\lambda t} \, \mathfrak {v}_n(t,x) \,dt \right] \\&= \lim _{R \rightarrow \infty } \left[ \frac{1}{\lambda } \mathfrak {v}_n(0,x) - \frac{e^{-\lambda R}}{\lambda }\mathfrak {v}_n(R,x) + \frac{1}{\lambda } \int _0^R e^{-\lambda t} (\tfrac{\partial }{\partial t}\mathfrak {v}_n)(t,x) \,dt \right] \\&= \frac{1}{\lambda } \left[ \mathfrak {v}_n(0,x) + \int _0^{\infty } e^{-\lambda t} (\tfrac{\partial }{\partial t}\mathfrak {v}_n)(t,x)\,dt \right] \\&= \frac{1}{\lambda } \left[ \mathfrak {h}_n(x) + \int _0^{\infty } e^{-\lambda t} \tfrac{1}{2}{\text {Trace}}(BB^{*}({\text {Hess}}_x \mathfrak {v}_n)(t,x))\,dt \right] \\&= \frac{1}{\lambda } \left[ \mathfrak {h}_n(x) + \tfrac{1}{2} {\text {Trace}}(BB^{*}({\text {Hess}} \mathfrak {u}_n)(x)) \right] \!. \end{aligned} \end{aligned}$$

This shows for every $n \in \mathbb {N}$ that $\mathfrak {u}_n$ is a viscosity solution of

31 $$\begin{aligned} \lambda \mathfrak {u}_n(x) - \tfrac{1}{2} {\text {Trace}}(BB^{*}({\text {Hess}} \mathfrak {u}_n)(x)) = \mathfrak {h}_n(x) \end{aligned}$$

for $x \in \mathbb {R}^d$. Next note that (26), (27), and (28) ensure for every non-empty compact set $K \subseteq \mathbb {R}^d\times \mathbb {R}\times \mathbb {R}^d \times \mathbb {S}_d$ that

32 $$\begin{aligned} \begin{aligned}&\limsup _{n \rightarrow \infty } \left[ \sup _{(x,r,p,A) \in K} \left| F_n(x,r,p,A) - F_0(x,r,p,A) \right| \right] \\&= \limsup _{n \rightarrow \infty } \left[ \sup _{(x,r,p,A) \in K} |\mathfrak {h}_n(x) - h(x)| \right] \\&\leqslant \limsup _{n \rightarrow \infty } \left( \left[ \sup _{y\in \mathbb {R}^d} \left( \frac{|\mathfrak {h}_n(y) - h(y)|}{V(y)} \right) \right] \left[ \sup _{(x,r,p,A) \in K} V(x) \right] \right) = 0. \end{aligned} \end{aligned}$$

Moreover, note that (23), (24), and (29) guarantee for all $n \in \mathbb {N}$, $x \in \mathbb {R}^d$ that

33 $$\begin{aligned} \begin{aligned}&\frac{ | \mathfrak {u}_n(x) - u(x) |}{ V(x) } = \frac{1}{V(x)} \left| \mathbb {E}\! \left[ \int _0^{\infty } e^{-\lambda s} \left( h(x+BW_s) - \mathfrak {h}_n(x+BW_s) \right) \!\,ds \right] \right| \\&\leqslant \int _0^{\infty } e^{-\lambda s} \left[ \sup _{y\in \mathbb {R}^d} \left( \frac{|h(y)-\mathfrak {h}_n(y)|}{V(y)} \right) \right] \frac{ \mathbb {E}\! \left[ V(x+BW_s) \right] }{V(x)} \, ds\\&\leqslant \frac{1}{\lambda -\rho } \left[ \sup _{y\in \mathbb {R}^d} \left( \frac{|h(y)-\mathfrak {h}_n(y)|}{V(y)} \right) \right] \!. \end{aligned} \end{aligned}$$

This and (26) imply for every non-empty compact set $K \subseteq \mathbb {R}^d$ that

34 $$\begin{aligned} \limsup _{n\rightarrow \infty } \left[ \sup _{x\in K} |\mathfrak {u}_n(x) - u(x) | \right] = 0. \end{aligned}$$

This, (32), the fact that for all $n \in \mathbb {N}$ it holds that $\mathfrak {u}_n$ is a viscosity solution of

35 $$\begin{aligned} F_n(x,\mathfrak {u}_n(x),(\nabla \mathfrak {u}_n)(x),({\text {Hess}} \mathfrak {u}_n)(x)) = 0 \end{aligned}$$

for $x \in \mathbb {R}^d$ (cf. (27) and (31)), and [36, Lemma 4.8] (see also [2]) imply that *u* is a viscosity solution of

36 $$\begin{aligned} F_0(x,u(x),(\nabla u)(x),({\text {Hess}} u)(x)) = 0 \end{aligned}$$

for $x \in \mathbb {R}^d$. This establishes (25). The proof of Lemma 2.4 is thus completed. $\square$

Unrolling the definition of viscosity solutions we can now infer from Lemma 2.4 above that solutions *u* to SFPEs in Corollary 2.2 are viscosity solutions of certain semilinear elliptic PDEs. Later on we will show that

viscosity solutions of the considered semilinear elliptic PDEs are unique in the same set of functions in which also uniqueness for the associated SFPEs has been established.

#### Corollary 2.5

Let $d,m \in \mathbb {N}$, $B \in \mathbb {R}^{d\times m}$, $L,\rho \in \mathbb {R}$, $\lambda \in (L+\rho ,\infty )$, let $\left\| \cdot \right\| :\mathbb {R}^d\rightarrow [0,\infty )$ be a norm on $\mathbb {R}^d$, let $f \in C( \mathbb {R}^d\times \mathbb {R},\mathbb {R})$, $u \in C(\mathbb {R}^d,\mathbb {R})$, $V \in C^2(\mathbb {R}^d,(0,\infty ))$ satisfy $\sup _{r\in (0,\infty )} [ \inf _{ \left\| x \right\| >r} V(x)] = \infty$ and $\inf _{r\in (0,\infty )} [ \sup _{ \left\| x \right\| >r} (\frac{|f(x,0)|+|u(x)|}{V(x)})] = 0$, assume for all $x \in \mathbb {R}^d$ that $\tfrac{1}{2} {\text {Trace}}\!\big ( BB^{*}({\text {Hess}} V)(x) \big ) \leqslant \rho V(x)$, let $( \Omega , \mathcal {F}, \mathbb {P})$ be a probability space, let $W :[0,\infty ) \times \Omega \rightarrow \mathbb {R}^m$ be a standard Brownian motion, and assume for all $x \in \mathbb {R}^d$, $v,w \in \mathbb {R}$ that $| f(x,v) - f(x,w) | \leqslant L |v-w|$ and

37 $$\begin{aligned} u(x) = \mathbb {E}\! \left[ \int _0^{\infty } e^{-\lambda t} f(x+BW_t,u(x+BW_t))\,dt \right] \end{aligned}$$

(cf. Corollary 2.2). Then it holds that *u* is a viscosity solution of

38 $$\begin{aligned} \lambda u(x) - \tfrac{1}{2} {\text {Trace}}\!\big (BB^{*}({\text {Hess}}u)(x)\big ) = f(x,u(x)) \end{aligned}$$

for $$ x \in \mathbb {R}^d $$.

#### Proof of Corollary 2.5

Corollary 2.5 is an immediate consequence of Lemma 2.4. A detailed proof can be found, e.g., in the extended preprint version [7, Corollary 2.11] of this article.

$$\square $$

### On a comparison principle for viscosity solutions of semilinear elliptic PDEs

In this subsection we prove a comparison principle for suitable viscosity solutions of certain semilinear elliptic PDEs; see Proposition 2.6 below. Combining this comparison principle with Corollary 2.2 and Corollary 2.5 will allow us to establish an existence, uniqueness, and Feynman–Kac type representation result for viscosity solutions of certain semilinear elliptic PDEs in Proposition 2.7 in Sect. 2.3 below. Our proof of the comparison principle Proposition 2.6 builds on [36] and [19] (see also [2] and [18]).

#### Proposition 2.6

(Comparison principle) Let $d,m \in \mathbb {N}$, $B \in \mathbb {R}^{d\times m}$, $L, \rho \in \mathbb {R}$, $\lambda \in (\rho +L,\infty )$, let $\left\| \cdot \right\| :\mathbb {R}^d\rightarrow [0,\infty )$ be the standard norm on $\mathbb {R}^d$, let $f \in C(\mathbb {R}^d\times \mathbb {R},\mathbb {R})$, $g,h,u,v \in C(\mathbb {R}^d,\mathbb {R})$, $V \in C^2(\mathbb {R}^d,(0,\infty ))$ satisfy for all $x\in \mathbb {R}^d$, $a,b\in \mathbb {R}$ that $[f(x,a)-f(x,b)-L(a-b)](a-b)\leqslant 0$ and

39 $$\begin{aligned} \tfrac{1}{2}{\text {Trace}}\!\left( BB^{*}({\text {Hess}} V)(x)\right) \leqslant \rho V(x), \end{aligned}$$

assume that $\limsup _{r\rightarrow \infty } [\sup _{ \left\| x \right\| >r} (\frac{|f(x,0)|+|g(x)|+|h(x)|+|u(x)|+|v(x)|}{V(x)})] = 0$, assume that *u* is viscosity supersolution of

40 $$\begin{aligned} \lambda u(x) - \tfrac{1}{2} {\text {Trace}}\!\left( BB^{*}({\text {Hess}} u)(x) \right) = f(x,u(x)) + g(x) \end{aligned}$$

for $x\in \mathbb {R}^d$, and assume that *v* is a viscosity subsolution of

41 $$\begin{aligned} \lambda v(x) - \tfrac{1}{2} {\text {Trace}}\!\left( BB^{*}({\text {Hess}} v)(x) \right) = f(x,v(x)) + h(x) \end{aligned}$$

for $$x\in \mathbb {R}^d$$. Then

42 $$\begin{aligned} \sup _{x\in \mathbb {R}^d} \left[ \max \left\{ \frac{v(x)-u(x)}{V(x)},0\right\} \right] \leqslant \frac{1}{\lambda -(L+\rho )} \left[ \sup _{x\in \mathbb {R}^d} \left( \max \left\{ \frac{h(x)-g(x)}{V(x)},0\right\} \right) \right] \!. \end{aligned}$$

#### Proof of Proposition 2.6

Throughout this proof let $\langle \cdot ,\cdot \rangle :\mathbb {R}^d\times \mathbb {R}^d\rightarrow \mathbb {R}$ be the standard scalar product on $\mathbb {R}^d$, let $w_1,w_2\in C(\mathbb {R}^d,\mathbb {R})$ satisfy for all $x\in \mathbb {R}^d$ that $w_1(x)=\frac{u(x)}{V(x)}$ and $w_2(x)=\frac{v(x)}{V(x)}$, and let $\eta _{\alpha }:\mathbb {R}^d\times \mathbb {R}^d\rightarrow \mathbb {R}$, $\alpha \in (0,\infty )$, satisfy for all $\alpha \in (0,\infty )$, $x,y\in \mathbb {R}^d$ that $\eta _{\alpha }(x,y) = w_2(x)-w_1(y)-\frac{\alpha }{2} \left\| x-y \right\| ^2$. Note that (42) is clear in the case of $v\leqslant u$. Therefore, we assume in the following that there exists $x\in \mathbb {R}^d$ such that $v(x)-u(x)>0$. This implies that there exists $x\in \mathbb {R}^d$ such that $w_2(x)-w_1(x)>0$. Next note that the hypothesis that $\limsup _{r\rightarrow \infty }[\sup _{ \left\| x \right\| >r} (\frac{|u(x)|+|v(x)|}{V(x)})] = 0$ demonstrates that

43 $$\begin{aligned} \limsup _{r\rightarrow \infty } \left[ \sup _{ \left\| x \right\| >r} \Big ( |w_1(x)|+|w_2(x)| \Big ) \right] = 0. \end{aligned}$$

Combining this with the fact that $w_1, w_2\in C(\mathbb {R}^d,\mathbb {R})$ and the fact that

44 $$\begin{aligned} \sup _{x\in \mathbb {R}^d} (\max \{w_2(x)-w_1(x),0\})\in (0,\infty ] \end{aligned}$$

implies that there exists $x_0\in \mathbb {R}^d$ which satisfies that

45 $$\begin{aligned} w_2(x_0)-w_1(x_0) = \sup _{y\in \mathbb {R}^d} \Big (w_2(y)-w_1(y)\Big ) > 0. \end{aligned}$$

In addition, note that (43) and the fact that $w_1, w_2 \in C(\mathbb {R}^d,\mathbb {R})$ ensure that $\sup _{x\in \mathbb {R}^d} (|w_1(x)|+|w_2(x)|) < \infty$. This and (45) imply for all $\alpha \in (0,\infty )$, $\beta \in (\alpha ,\infty )$ that

46 $$\begin{aligned} \begin{aligned} \infty&> \left[ \sup _{ x \in \mathbb {R}^d } |w_1(x)| \right] + \left[ \sup _{ x \in \mathbb {R}^d } |w_2(x)| \right] \geqslant \sup _{ x,y \in \mathbb {R}^d } (w_2(x) - w_1(y)) \\  &\geqslant \sup _{ z \in \mathbb {R}^d \times \mathbb {R}^d } \eta _{\alpha }(z) \geqslant \sup _{ z \in \mathbb {R}^d \times \mathbb {R}^d } \eta _{\beta }(z) \geqslant \sup _{ x \in \mathbb {R}^d } \eta _{\beta }(x,x) \\  &= \sup _{ x \in \mathbb {R}^d } (w_2(x)-w_1(x)) > 0. \end{aligned} \end{aligned}$$

In the next step let $r_{\alpha } \in (0,\infty )$, $\alpha \in (0,\infty )$, satisfy for all $\alpha \in (0,\infty )$ that  and let $R \in (0,\infty )$ satisfy that for all $x \in \mathbb {R}^d$ with $\left\| x \right\| >R$ it holds that

47 $$\begin{aligned} \textstyle |w_1(x)| + |w_2(x)| < \frac{1}{4} \sup _{y\in \mathbb {R}^d} (w_2(y)-w_1(y)). \end{aligned}$$

Furthermore, observe that for all $\alpha \in (0,\infty )$, $x,y \in \mathbb {R}^d$ with $\left\| x-y \right\| > r_{\alpha }$ it holds that $w_2(x)-w_1(y)-\frac{\alpha }{2} \left\| x-y \right\| ^2 \leqslant 0$. Hence, we obtain for all $\alpha \in (0,\infty )$, $x,y \in \mathbb {R}^d$ with $\max \{ \left\| x \right\| , \left\| y \right\| \} > R+r_{\alpha }$ that

48 $$\begin{aligned} \eta _{\alpha }(x,y) {\left\{ \begin{array}{ll} \leqslant 0 &  : \left\| x-y \right\| > r_{\alpha }, \\ \leqslant \frac{1}{2} \sup _{z\in \mathbb {R}^d} (w_2(z)-w_1(z)) &  : \left\| x-y \right\| \leqslant r_{\alpha }. \end{array}\right. } \end{aligned}$$

This and (46) show for all $\alpha \in (0,\infty )$ that

49 $$\begin{aligned} \sup _{z\in \mathbb {R}^d\times \mathbb {R}^d} \eta _{\alpha }(z) = \sup _{\max \{ \left\| x \right\| , \left\| y \right\| \}\leqslant R+r_{\alpha }} \eta _{\alpha }(x,y). \end{aligned}$$

Hence, we obtain that for every $\alpha \in (0,\infty )$ there exist $x_{\alpha },y_{\alpha }\in \mathbb {R}^d$ which satisfy that $\max \{ \left\| x_{\alpha } \right\| , \left\| y_{\alpha } \right\| \} \leqslant R + r_{\alpha }$ and

50 $$\begin{aligned} w_2(x_{\alpha })-w_1(y_{\alpha })- \frac{\alpha }{2} \left\| x_{\alpha }-y_{\alpha } \right\| ^2 = \sup _{z\in \mathbb {R}^d\times \mathbb {R}^d} \eta _{\alpha }(z) > 0. \end{aligned}$$

[19, Theorem 3.2] (with $k \leftarrow 2$, $N_1 \leftarrow d$, $N_2 \leftarrow d$, $\mathcal {O}_1 \leftarrow \mathbb {R}^d$, $\mathcal {O}_2 \leftarrow \mathbb {R}^d$, $u_1 \leftarrow w_2$, $u_2 \leftarrow -w_1$, $\varphi \leftarrow (\mathbb {R}^d\times \mathbb {R}^d\ni (x,y)\mapsto \frac{\alpha }{2} \left\| x-y \right\| ^2\in \mathbb {R})$, $\hat{x} \leftarrow (x_{\alpha },y_{\alpha })$ for $\alpha \in (0,\infty )$ in the notation of [19, Theorem 3.2]) therefore guarantees that there exist $X_{\alpha },Y_{\alpha }\in \mathbb {S}_{d}$, $\alpha \in (0,\infty )$, which satisfy for all $\alpha \in (0,\infty )$ that $(\alpha (x_{\alpha }-y_{\alpha }),X_{\alpha }) \in (\hat{J}^{2}_{+}w_2)(x_{\alpha })$, $(\alpha (x_{\alpha }-y_{\alpha }),Y_{\alpha }) \in (\hat{J}^{2}_{-}w_1)(y_{\alpha })$, and

51 $$\begin{aligned} -3 \alpha \begin{pmatrix} I & \quad 0 \\ 0 & \quad I \end{pmatrix} \leqslant \begin{pmatrix} X_{\alpha } & \quad 0 \\ 0 & \quad -Y_{\alpha } \end{pmatrix} \leqslant 3 \alpha \begin{pmatrix} I & \quad -I \\ -I & \quad I \end{pmatrix} \end{aligned}$$

(see [36, Definition 4.3] for definitions of $\hat{J}^{2}_{+}w_2$ and $\hat{J}^{2}_{-}w_1$). Next observe that (40) implies that $w_1$ is a viscosity supersolution of

52 $$\begin{aligned} \begin{aligned}&\lambda w_1(x) - \bigg [ \tfrac{1}{2}{\text {Trace}}\left( BB^{*}({\text {Hess}} w_1)(x) \right) + \left\langle BB^{*}\tfrac{(\nabla V)(x)}{V(x)}, (\nabla w_1)(x) \right\rangle \\&+ \tfrac{1}{2}{\text {Trace}}\!\left( BB^{*}\tfrac{({\text {Hess}} V)(x)}{V(x)} \right) w_1(x) + \tfrac{1}{V(x)}f\big (x,V(x)w_1(x)\big ) + \tfrac{g(x)}{V(x)} \bigg ] = 0 \end{aligned} \end{aligned}$$

for $x\in \mathbb {R}^d$. Combining this and (51) assures for all $\alpha \in (0,\infty )$ that

53 $$\begin{aligned} \begin{aligned}&\lambda w_1(y_{\alpha }) - \bigg [ \tfrac{1}{2}{\text {Trace}}\left( BB^{*}Y_{\alpha } \right) + \left\langle BB^{*}\tfrac{(\nabla V)(y_{\alpha })}{V(y_{\alpha })}, \alpha (x_{\alpha }-y_{\alpha }) \right\rangle \\&+ \tfrac{1}{2}{\text {Trace}}\!\left( BB^{*}\tfrac{({\text {Hess}} V)(y_{\alpha })}{V(y_{\alpha })} \right) w_1(y_{\alpha }) + \tfrac{1}{V(y_{\alpha })}f\big (y_{\alpha },V(y_{\alpha })w_1(y_{\alpha })\big ) + \tfrac{g(y_{\alpha })}{V(y_{\alpha })} \bigg ] \\  &\geqslant 0. \end{aligned} \end{aligned}$$

In addition, note that (41) ensures that $w_2$ is a viscosity subsolution of

54 $$\begin{aligned} \begin{aligned}&\lambda w_2(x) - \bigg [ \tfrac{1}{2}{\text {Trace}}\left( BB^{*}({\text {Hess}} w_2)(x) \right) + \left\langle BB^{*}\tfrac{(\nabla V)(x)}{V(x)}, (\nabla w_2)(x) \right\rangle \\&+ \tfrac{1}{2}{\text {Trace}}\!\left( BB^{*}\tfrac{({\text {Hess}} V)(x)}{V(x)} \right) w_2(x) + \tfrac{1}{V(x)}f\big (x,V(x)w_2(x)\big ) + \tfrac{h(x)}{V(x)} \bigg ] = 0 \end{aligned} \end{aligned}$$

for $x \in \mathbb {R}^d$. Combining this and (51) implies for all $\alpha \in (0,\infty )$ that

55 $$\begin{aligned} \begin{aligned}&\lambda w_2(x_{\alpha }) - \bigg [ \tfrac{1}{2}{\text {Trace}}\left( BB^{*}X_{\alpha } \right) + \left\langle BB^{*}\tfrac{(\nabla V)(x_{\alpha })}{V(x_{\alpha })}, \alpha (x_{\alpha }-y_{\alpha }) \right\rangle \\&+ \tfrac{1}{2}{\text {Trace}}\!\left( BB^{*}\tfrac{({\text {Hess}} V)(x_{\alpha })}{V(x_{\alpha })} \right) w_2(x_{\alpha }) + \tfrac{1}{V(x_{\alpha })}f\big (x_{\alpha },V(x_{\alpha })w_2(x_{\alpha })\big ) + \tfrac{h(x_{\alpha })}{V(x_{\alpha })} \bigg ] \\  &\leqslant 0. \end{aligned} \end{aligned}$$

This and (53) assure for all $\alpha \in (0,\infty )$ that

56 $$\begin{aligned} \begin{aligned}&\lambda (w_2(x_{\alpha }) - w_1(y_{\alpha })) \\  &\leqslant \tfrac{1}{2} {\text {Trace}}\!\left( BB^{*}(X_{\alpha }-Y_{\alpha }) \right) + \left\langle BB^{*}\left( \tfrac{(\nabla V)(x_{\alpha })}{V(x_{\alpha })} - \tfrac{(\nabla V)(y_{\alpha })}{V(y_{\alpha })}\right) , \alpha (x_{\alpha }-y_{\alpha }) \right\rangle \\&+ \tfrac{1}{2} {\text {Trace}}\!\left( BB^{*}\left( \tfrac{({\text {Hess}} V)(x_{\alpha })}{V(x_{\alpha })} w_2(x_{\alpha }) - \tfrac{({\text {Hess}} V)(y_{\alpha })}{V(y_{\alpha })} w_1(y_{\alpha })\right) \right) \\&+ \tfrac{1}{V(x_{\alpha })}f\big (x_{\alpha },V(x_{\alpha })w_2(x_{\alpha })\big ) - \tfrac{1}{V(y_{\alpha })}f\big (y_{\alpha },V(y_{\alpha })w_1(y_{\alpha })\big ) + \tfrac{h(x_{\alpha })}{V(x_{\alpha })}-\tfrac{g(y_{\alpha })}{V(y_{\alpha })}. \end{aligned} \end{aligned}$$

Next note that (51) ensures for all $\alpha \in (0,\infty )$ that $X_{\alpha } \leqslant Y_{\alpha }$. This and (56) imply for all $\alpha \in (0,\infty )$ that

57 $$\begin{aligned}&\lambda (w_2(x_{\alpha })-w_1(y_{\alpha }) ) \leqslant \left\langle BB^{*}\left( \tfrac{(\nabla V)(x_{\alpha })}{V(x_{\alpha })} - \tfrac{(\nabla V)(y_{\alpha })}{V(y_{\alpha })}\right) , \alpha (x_{\alpha }-y_{\alpha }) \right\rangle \nonumber \\&+ \tfrac{1}{2} {\text {Trace}}\!\left( BB^{*}\!\left( \tfrac{({\text {Hess}} V)(x_{\alpha })}{V(x_{\alpha })} w_2(x_{\alpha }) - \tfrac{({\text {Hess}} V)(y_{\alpha })}{V(y_{\alpha })} w_1(y_{\alpha })\right) \right) \nonumber \\&+ \tfrac{1}{V(x_{\alpha })}f\big (x_{\alpha },V(x_{\alpha })w_2(x_{\alpha })\big ) - \tfrac{1}{V(y_{\alpha })}f\big (y_{\alpha },V(y_{\alpha })w_1(y_{\alpha })\big ) + \tfrac{h(x_{\alpha })}{V(x_{\alpha })}-\tfrac{g(y_{\alpha })}{V(y_{\alpha })}. \end{aligned}$$

Moreover, observe that (46) implies that $\lim _{\alpha \rightarrow \infty } [\sup _{z\in \mathbb {R}^d\times \mathbb {R}^d} \eta _{\alpha }(z)] \in \mathbb {R}$ exists. [36, Lemma 4.9] (with $d \leftarrow 2d$, $O \leftarrow \mathbb {R}^d\times \mathbb {R}^d$, $\eta \leftarrow ( \mathbb {R}^d\times \mathbb {R}^d \ni (x,y) \mapsto w_2(x) - w_1(y) \in \mathbb {R})$, $\phi \leftarrow ( \mathbb {R}^d\times \mathbb {R}^d \ni (x,y) \mapsto \frac{1}{2} \left\| x-y \right\| ^2 \in [0,\infty ) )$, $x \leftarrow ( (0,\infty ) \ni \alpha \mapsto (x_{\alpha },y_{\alpha }) \in \mathbb {R}^d\times \mathbb {R}^{d} )$ in the notation of [36, Lemma 4.9]) and (50) therefore ensure that $\limsup _{\alpha \rightarrow \infty } [\alpha \left\| x_{\alpha }-y_{\alpha } \right\| ^2] = 0$. This, the fact that $\limsup _{\alpha \rightarrow \infty } r_{\alpha } = 0$, the fact that $\frac{\nabla V}{V} :\mathbb {R}^d \rightarrow \mathbb {R}^d$ is locally Lipschitz continuous, and (50) imply that

58 $$\begin{aligned} \limsup _{\alpha \rightarrow \infty } \left| \left\langle BB^{*}\!\left( \tfrac{(\nabla V)(x_{\alpha })}{V(x_{\alpha })} - \tfrac{(\nabla V)(y_{\alpha })}{V(y_{\alpha })} \right) , \alpha (x_{\alpha }-y_{\alpha }) \right\rangle \right| = 0. \end{aligned}$$

In addition, note that the fact that $\limsup _{\alpha \rightarrow \infty } r_{\alpha } = 0$ and (50) assure that there exist $\hat{x} \in \mathbb {R}^d$ and $\alpha _n \in (0,\infty )$, $n\in \mathbb {N}$, which satisfy that $\liminf _{n\rightarrow \infty } \alpha _n=\infty$ and $\limsup _{n\rightarrow \infty } \left\| x_{\alpha _{n}}-\hat{x} \right\| = 0$. This, the fact that $({\text {Hess}} V) \in C(\mathbb {R}^d,\mathbb {S}_d)$, the fact that $f \in C(\mathbb {R}^d \times \mathbb {R}, \mathbb {R})$, the fact that $V \in C(\mathbb {R}^d, (0,\infty ) )$, the fact that $u, v, g, h \in C(\mathbb {R}^d,\mathbb {R})$, and the fact that $\limsup _{\alpha \rightarrow \infty } [\frac{\alpha }{2} \left\| x_{\alpha }-y_{\alpha } \right\| ^2] = 0$ prove that

(i) it holds that
  59 $$\begin{aligned} \begin{aligned}&\limsup _{n\rightarrow \infty } \left[ \biggl | \tfrac{1}{2} {\text {Trace}}\!\left( BB^{*} \tfrac{({\text {Hess}} V)(x_{\alpha _n})}{V(x_{\alpha _n})}w_2(x_{\alpha _n}) \right) \right. \\  &- \tfrac{1}{2} {\text {Trace}}\!\left( BB^{*} \tfrac{({\text {Hess}} V)(\hat{x})}{V(\hat{x})} w_2(\hat{x}) \right) \biggr | \\&+ \biggl | \tfrac{1}{2} {\text {Trace}}\!\left( BB^{*} \tfrac{({\text {Hess}} V)(y_{\alpha _n})}{V(y_{\alpha _n})}w_1(y_{\alpha _n}) \right) \\  &\left. - \tfrac{1}{2} {\text {Trace}}\!\left( BB^{*} \tfrac{({\text {Hess}} V)(\hat{x})}{V(\hat{x})} w_1(\hat{x}) \right) \biggr |\right] = 0, \end{aligned} \end{aligned}$$
(ii) it holds that
  60 $$\begin{aligned} \begin{aligned}&\limsup _{n\rightarrow \infty } \biggl [ \left| \tfrac{1}{V(x_{\alpha _n})} f\big (x_{\alpha _n},V(x_{\alpha _n})w_2(x_{\alpha _n})\big ) - \tfrac{1}{V(\hat{x})} f\big (\hat{x},V(\hat{x})w_2(\hat{x})\big ) \right| \\&+ \left| \tfrac{1}{V(y_{\alpha _n})} f\big (y_{\alpha _n},V(y_{\alpha _n})w_1(y_{\alpha _n})\big ) - \tfrac{1}{V(\hat{x})} f\big (\hat{x},V(\hat{x})w_1(\hat{x})\big ) \right| \biggr ] = 0, \end{aligned} \end{aligned}$$
  and
(iii) it holds that
  61 $$\begin{aligned} \limsup _{n\rightarrow \infty } \biggl [ \Bigl | \tfrac{h(x_{\alpha _n})}{V(x_{\alpha _n})} - \tfrac{h(\hat{x})}{V(\hat{x})} \Bigr | + \Bigl | \tfrac{g(y_{\alpha _n})}{V(y_{\alpha _n})} - \tfrac{g(\hat{x})}{V(\hat{x})} \Bigr | \biggr ] = 0. \end{aligned}$$

Combining this with (57) and (58) shows that

62 $$\begin{aligned} \begin{aligned}&\lambda ( w_2(\hat{x}) - w_1(\hat{x}) ) \\&\leqslant \tfrac{1}{2} {\text {Trace}}\!\left( BB^{*}\tfrac{({\text {Hess}} V)(\hat{x})}{V(\hat{x})} \right) (w_2(\hat{x})-w_1(\hat{x})) \\&+ \tfrac{f(\hat{x},V(\hat{x})w_2(\hat{x}))-f(\hat{x},V(\hat{x})w_1(\hat{x}))}{V(\hat{x})} + \tfrac{h(\hat{x})}{V(\hat{x})} - \tfrac{g(\hat{x})}{V(\hat{x})}. \end{aligned} \end{aligned}$$

Next note that the second part of the statement of [36, Lemma 4.9] (with $d \leftarrow 2d$, $O \leftarrow \mathbb {R}^d\times \mathbb {R}^d$, $\eta \leftarrow ( \mathbb {R}^d\times \mathbb {R}^d \ni (x,y) \mapsto w_2(x) - w_1(y) \in \mathbb {R})$, $\phi \leftarrow ( \mathbb {R}^d\times \mathbb {R}^d \ni (x,y) \mapsto \frac{1}{2} \left\| x-y \right\| ^2 \in [0,\infty ) )$, $x \leftarrow ( (0,\infty ) \ni \alpha \mapsto (x_{\alpha },y_{\alpha }) \in \mathbb {R}^d\times \mathbb {R}^{d} )$, $(\alpha _n)_{n\in \mathbb {N}} \leftarrow (\alpha _n)_{n\in \mathbb {N}}$, $x_0 \leftarrow (\hat{x},\hat{x})$ in the notation of [36, Lemma 4.9]) demonstrates that $w_2(\hat{x})-w_1(\hat{x}) = \sup _{x\in \mathbb {R}^d} (w_2(x)-w_1(x)) > 0$. This, (62), (39), and the assumption that for all $x \in \mathbb {R}^d$, $a,b \in \mathbb {R}$ it holds that $[f(x,a)-f(x,b)](a-b) \leqslant L |a-b|^2$ ensure that

63 $$\begin{aligned} \begin{aligned}&\lambda \left[ \sup _{z\in \mathbb {R}^d} ( \max \{ w_2(z)-w_1(z), 0 \} ) \right] \\&= \lambda \left( w_2(\hat{x}) - w_1(\hat{x}) \right) \leqslant \rho \left( w_2(\hat{x})-w_1(\hat{x})\right) \\&+ \tfrac{ L [V(\hat{x})w_2(\hat{x}) - V(\hat{x})w_1(\hat{x})]}{V(\hat{x})} + \tfrac{h(\hat{x})}{V(\hat{x})} - \tfrac{g(\hat{x})}{V(\hat{x})} \\&\leqslant (\rho + L) (w_2(\hat{x})-w_1(\hat{x})) + \sup _{z\in \mathbb {R}^d} \left[ \max \left\{ \tfrac{h(z)-g(z)}{V(z)},0 \right\} \right] \\&= (\rho + L) \left[ \sup _{z\in \mathbb {R}^d} ( \max \{ w_2(z)-w_1(z), 0 \} ) \right] + \sup _{z\in \mathbb {R}^d} \left[ \max \left\{ \tfrac{h(z)-g(z)}{V(z)},0 \right\} \right] \!. \end{aligned} \end{aligned}$$

Hence, we obtain that

64 $$\begin{aligned} {[}\lambda - (L+\rho ){]} \biggl [ \sup _{x\in \mathbb {R}^d}( \max \{\tfrac{v(x) - u(x)}{V(x)},0\}) \biggr ] \leqslant \sup _{x\in \mathbb {R}^d}[\max \{ \tfrac{h(x)-g(x)}{V(x)},0 \} ]. \end{aligned}$$

This establishes (42). The proof of Proposition 2.6 is thus completed. $\square$

### Existence and uniqueness results for viscosity solutions of semilinear elliptic PDEs

In this subsection we establish stochastic representation formulas of the Feynman–Kac type for suitable viscosity solutions of certain semilinear elliptic PDEs, see Proposition 2.7, Corollary 2.8, and Corollary 2.10 below.

By combining the existence and uniqueness result for SFPEs in Corollary 2.2 with the Feynman–Kac type representation result in Corollary 2.5 and the comparison principle in Proposition 2.6 we obtain the following existence, uniqueness and Feynman–Kac type representation result for viscosity solutions of certain semilinear elliptic PDEs in Proposition 2.7.

#### Proposition 2.7

(Existence and uniqueness of viscosity solutions) Let $d,m\in \mathbb {N}$, $B\in \mathbb {R}^{d\times m}$, $L,\rho \in \mathbb {R}$, $\lambda \in (\rho +L,\infty )$, let $\left\| \cdot \right\| :\mathbb {R}^d\rightarrow [0,\infty )$ be a norm on $\mathbb {R}^d$, let $f\in C(\mathbb {R}^d\times \mathbb {R},\mathbb {R})$, $V\in C^2(\mathbb {R}^d,(0,\infty ))$ satisfy for all $x\in \mathbb {R}^d$, $v,w\in \mathbb {R}$ that $|f(x,v)-f(x,w)|\leqslant L|v-w|$ and

65 $$\begin{aligned} \tfrac{1}{2}{\text {Trace}}\!\left( BB^{*}({\text {Hess}} V)(x)\right) \leqslant \rho V(x), \end{aligned}$$

assume that $\inf _{r\in (0,\infty )} [\sup _{ \left\| x \right\| >r} (\frac{|f(x,0)|}{V(x)})] = 0$ and $\sup _{r\in (0,\infty )} [ \inf _{ \left\| x \right\| >r} V(x) ] = \infty$, let $( \Omega , \mathcal {F}, \mathbb {P})$ be a probability space, and let $W :[0,\infty ) \times \Omega \rightarrow \mathbb {R}^m$ be a standard Brownian motion. Then

(i) there exists a unique $u \in \{ v \in C(\mathbb {R}^d,\mathbb {R}):\inf _{r\in (0,\infty )} [\sup _{ \left\| x \right\| >r}(\frac{|v(x)|}{V(x)})] = 0 \}$ which satisfies that *u* is a viscosity solution of
  66 $$\begin{aligned} \lambda u( x ) - \tfrac{1}{2} {\text {Trace}}\!\big (BB^{*}({\text {Hess}}u)(x)\big ) = f(x,u(x)) \end{aligned}$$
  for $$x\in \mathbb {R}^d$$ and
(ii) it holds for all $x \in \mathbb {R}^d$ that $\mathbb {E}\big [ \int _0^{\infty } e^{-\lambda t} |f(x+BW_t,u(x+BW_t))|\,dt \big ] < \infty$ and
  67 $$\begin{aligned} u(x) = \mathbb {E}\! \left[ \int _0^{\infty } e^{-\lambda t} f(x+BW_t,u(x+BW_t))\,dt \right] \!. \end{aligned}$$

#### Proof of Proposition 2.7

Proposition 2.7 is a direct consequence of Corollary 2.2, Corollary 2.5, and Proposition 2.6. We refer, e.g., to the extended preprint version [7, Proposition 2.13] of this article for more details. $\square$

In Corollary 2.8 below we reformulate Proposition 2.7 for the setting in which the semilinear elliptic PDE under consideration is given in a slightly different form.

#### Corollary 2.8

Let $d,m \in \mathbb {N}$, $B \in \mathbb {R}^{d\times m}$, $L, \rho \in \mathbb {R}$, $\lambda \in (\rho + L,\infty )$, let $\left\| \cdot \right\| :\mathbb {R}^d \rightarrow [0,\infty )$ be a norm on $\mathbb {R}^d$, let $f \in C(\mathbb {R}^d\times \mathbb {R},\mathbb {R})$, $V \in C^2(\mathbb {R}^d,(0,\infty ))$ satisfy for all $x \in \mathbb {R}^d$, $v,w \in \mathbb {R}$ that $| f(x,v) - f(x,w) - \lambda (v-w) | \leqslant L | v-w |$ and $\frac{1}{2} {\text {Trace}}(BB^{*}({\text {Hess }}V)(x)) \leqslant \rho V(x)$, assume that $\inf _{r\in (0,\infty )} [\sup _{ \left\| x \right\| >r} (\frac{|f(x,0)|}{V(x)})] = 0$ and $\sup _{r\in (0,\infty )} [\inf _{ \left\| x \right\| >r} V(x)] = \infty$, let $(\Omega ,\mathcal {F},\mathbb {P})$ be a probability space, and let $W :[0,\infty ) \times \Omega \rightarrow \mathbb {R}^m$ be a standard Brownian motion. Then

(i) there exists a unique $u \in \{ v \in C(\mathbb {R}^d,\mathbb {R}) :\inf _{r\in (0,\infty )} [\sup _{ \left\| x \right\| >r}(\frac{|v(x)|}{V(x)})] = 0 \}$ which satisfies that *u* is a viscosity solution of
  68 $$\begin{aligned} \tfrac{1}{2} {\text {Trace}}\!\big (BB^{*}({\text {Hess}}u)(x)\big )=f(x,u(x)) \end{aligned}$$
  for $$x\in \mathbb {R}^d$$ and
(ii) it holds for all $x \in \mathbb {R}^d$ that $\mathbb {E}\big [ \int _0^{\infty } e^{-\lambda t} | \lambda u(x+BW_t) - f(x+BW_t,u(x+BW_t)) | \,dt \big ] < \infty$ and
  69 $$\begin{aligned} u(x) = \mathbb {E}\! \left[ \int _0^{\infty } e^{-\lambda t} ( \lambda u(x+BW_t) - f(x+BW_t,u(x+BW_t)) ) \,dt \right] \!. \end{aligned}$$

#### Proof of Corollary 2.8

Corollary 2.8 is an immediate consequence of Proposition 2.7. We refer, e.g., to the extended preprint version [7, Corollary 2.14] of this article for more details. $\square$

In Lemma 2.9 below we carry out some elementary auxiliary calculations for a special choice of Lyapunov functions which will be frequently used later on.

#### Lemma 2.9

Let $d,m \in \mathbb {N}$, $B \in \mathbb {R}^{d\times m}$, $\varepsilon \in (0,\infty )$, $\left\| \cdot \right\| :(\cup _{k\in \mathbb {N}} \mathbb {R}^k) \rightarrow [0,\infty )$ satisfy for all $k \in \mathbb {N}$, $x = (x_1,\ldots ,x_k) \in \mathbb {R}^k$ that  and let $V :\mathbb {R}^d \rightarrow (0,\infty )$ satisfy for all $x \in \mathbb {R}^d$ that70Then it holds for all $x \in \mathbb {R}^d$ that

71 $$\begin{aligned} \frac{ \left\| B^{*}(\nabla V)(x) \right\| ^2}{V(x)} \leqslant \varepsilon ^2 \left[ \sup _{y\in \mathbb {R}^m{\setminus }\{0\}} \left( \frac{ \left\| By \right\| }{ \left\| y \right\| }\right) \right] ^2 V(x) \end{aligned}$$

and

72 $$\begin{aligned} \begin{aligned} {\text {Trace}}\!\big ( BB^{*}({\text {Hess}} V)(x) \big )&\leqslant (\varepsilon ^2 + \varepsilon d) \left[ \sup _{y\in \mathbb {R}^m{\setminus }\{0\}} \left( \frac{ \left\| By \right\| }{ \left\| y \right\| }\right) \right] ^2 V(x). \end{aligned} \end{aligned}$$

#### Proof of Lemma 2.9

Throughout this proof let $\Vert \!| \cdot \Vert \!| :\mathbb {R}^{d\times m} \rightarrow [0,\infty )$ be the Frobenius norm on $\mathbb {R}^{d\times m}$. Observe that for all $x \in \mathbb {R}^d$ it holds that73Hence, we obtain for all $x \in \mathbb {R}^d$ that

74 $$\begin{aligned} \frac{ \left\| B^{*}(\nabla V)(x) \right\| ^2}{V(x)} = \frac{\varepsilon ^2 \left\| B^{*}x \right\| ^2}{1+ \left\| x \right\| ^2} V(x) \leqslant \varepsilon ^2 \left[ \sup _{y\in \mathbb {R}^m{\setminus }\{0\}} \left( \frac{ \left\| By \right\| }{ \left\| y \right\| }\right) \right] ^2 V(x). \end{aligned}$$

Moreover, note that (73) demonstrates for all $x \in \mathbb {R}^d$ that75The proof of Lemma 2.9 is thus completed. $\square$

In Corollary 2.10 below we state a version of an existence, uniqueness, and Feynman–Kac type representation result for viscosity solutions of semilinear elliptic PDEs with a concrete choice of Lyapunov function. Corollary 2.10 follows by combining Corollary 2.8 and Lemma 2.9.

#### Corollary 2.10

Let $d,m \in \mathbb {N}$, $B \in \mathbb {R}^{d\times m}$, $L \in \mathbb {R}$, $\lambda \in (L,\infty )$, $f \in C(\mathbb {R}^d\times \mathbb {R},\mathbb {R})$ satisfy for all $x \in \mathbb {R}^d$, $v,w \in \mathbb {R}$ that $| f(x,v) - f(x,w) - \lambda ( v - w ) | \leqslant L | v-w |$, assume that *f* is at most polynomially growing, let $(\Omega ,\mathcal {F},\mathbb {P})$ be a probability space, and let $W :[0,\infty ) \times \Omega \rightarrow \mathbb {R}^m$ be a standard Brownian motion. Then

(i) there exists a unique
  76 $$\begin{aligned}  &   \textstyle u \in \bigl \{ v \in C(\mathbb {R}^d,\mathbb {R}) :\nonumber \\  &   \quad \quad \quad \quad \quad \qquad \textstyle (\forall \,\varepsilon \in (0,\infty ):\bigl [ \sup _{x=(x_1,\ldots ,x_d)\in \mathbb {R}^d} (\frac{|v(x)|}{\exp (\varepsilon \sum _{i=1}^d |x_i|)}) \bigr ] < \infty ) \bigr \} \end{aligned}$$
  which satisfies that *u* is a viscosity solution of
  77 $$\begin{aligned} \tfrac{1}{2}{\text {Trace}}\!\big (BB^{*}({\text {Hess}} u)(x)\big ) = f(x,u(x)) \end{aligned}$$
  for $$ x \in \mathbb {R}^d $$ and
(ii) it holds for all $x \in \mathbb {R}^d$ that $\mathbb {E}\big [ \int _0^{\infty } e^{-\lambda t} | \lambda u(x+BW_t) - f(x+BW_t,u(x+BW_t)) | \,dt \big ] < \infty$ and
  78 $$\begin{aligned} u(x) = \mathbb {E}\! \left[ \int _0^{\infty } e^{-\lambda t} ( \lambda u(x+BW_t) - f(x+BW_t,u(x+BW_t)) ) \,dt \right] \!. \end{aligned}$$

#### Proof of Corollary 2.10

Throughout this proof let $\left\| \cdot \right\| :\mathbb {R}^d \rightarrow [0,\infty )$ be the standard norm on $\mathbb {R}^d$, let $\beta , p \in [0,\infty )$ satisfy $\sup _{x\in \mathbb {R}^d} \left( \frac{|f(x,0)|}{1+ \left\| x \right\| ^p}\right) < \infty$ and $\beta = \tfrac{1}{2} \Bigg [\sup _{x=(x_1,\ldots ,x_m)\in \mathbb {R}^m{{\setminus }}\{0\}}\Bigg (\frac{ \left\| Bx \right\| ^2}{\sum _{i=1}^m |x_i|^2}\Bigg )\Bigg ]$, and let $V_{\varepsilon } :\mathbb {R}^d \rightarrow (0,\infty )$, $\varepsilon \in (0,\infty )$, satisfy for all $\varepsilon \in (0,\infty )$, $x \in \mathbb {R}^d$ that . Observe that Lemma 2.9 demonstrates for all $\varepsilon \in (0,\infty )$, $x \in \mathbb {R}^d$ that

79 $$\begin{aligned} \tfrac{1}{2}{\text {Trace}}\!\big ( BB^{*} ({\text {Hess}} V_{\varepsilon })(x) \big ) \leqslant (\varepsilon ^2+\varepsilon d)\beta V_{\varepsilon }(x). \end{aligned}$$

In addition, note that for all $\varepsilon \in (0,\infty )$, $x \in \mathbb {R}^d$ it holds that

80 $$\begin{aligned} \exp (\varepsilon \Vert x \Vert ) \leqslant V_{\varepsilon }(x) \leqslant e^{\varepsilon } \exp (\varepsilon \Vert x \Vert ). \end{aligned}$$

Moreover, note that for all $\varepsilon \in (0,\infty )$ it holds that $\sup _{r\in (0,\infty )} [ \inf _{ \left\| x \right\| >r} V_{\varepsilon }(x) ] = \infty$ and

81 $$\begin{aligned} \begin{aligned}&\limsup _{r\rightarrow \infty } \left[ \sup _{ \left\| x \right\|>r} \left( \frac{|f(x,0)|}{V_{\varepsilon }(x)} \right) \right] \\  &\leqslant \left[ \sup _{x\in \mathbb {R}^d} \left( \frac{|f(x,0)|}{1+ \left\| x \right\| ^p} \right) \right] \left[ \limsup _{r\rightarrow \infty } \left( \sup _{ \left\| x \right\| >r} \left( \frac{1+ \left\| x \right\| ^p}{\exp (\varepsilon \Vert x \Vert )} \right) \right) \right] = 0. \end{aligned} \end{aligned}$$

This, (79), (80), and Corollary 2.8 ensure for every $\varepsilon \in (0,\infty )$ with $(\varepsilon ^2+\varepsilon d) \beta < \lambda - L$ that there exists a unique $u_{\varepsilon } \in \{ v \in C(\mathbb {R}^d,\mathbb {R}) :\inf _{r\in (0,\infty )} [\sup _{ \left\| x \right\| >r} (\frac{|v(x)|}{V_{\varepsilon }(x)})] = 0 \}$ which satisfies that $u_{\varepsilon }$ is a viscosity solution of

82 $$\begin{aligned} \tfrac{1}{2} {\text {Trace}}(BB^{*}({\text {Hess}} u_{\varepsilon })(x)\big ) = f(x,u_{\varepsilon }(x)) \end{aligned}$$

for $x\in \mathbb {R}^d$. Corollary 2.8 hence assures for all $\varepsilon \in (0,\infty )$, $x \in \mathbb {R}^d$ with $(\varepsilon ^2+\varepsilon d) \beta < \lambda - L$ that

83 $$\begin{aligned} u_{\varepsilon }(x) = \mathbb {E}\! \left[ \int _0^{\infty } e^{-\lambda t} ( \lambda u_{\varepsilon }(x+BW_t) - f(x+BW_t,u_{\varepsilon }(x+BW_t)) ) \,dt \right] \!. \end{aligned}$$

Next note that the fact that for all $\varepsilon \in (0,\infty )$, $\eta \in (0,\varepsilon )$, $x \in \mathbb {R}^d$ it holds that $V_{\eta }(x) \leqslant V_{\varepsilon }(x)$ and (82) ensure that for all $\varepsilon \in (0,\infty )$, $\eta \in (0,\varepsilon )$, $x \in \mathbb {R}^d$ with $(\varepsilon ^2+\varepsilon d) \beta < \lambda - L$ it holds that $u_{\varepsilon }(x) = u_{\eta }(x)$. Hence, we obtain that there exists $\mathfrak {u} :\mathbb {R}^d \rightarrow \mathbb {R}$ which satisfies for all $\varepsilon \in (0,\infty )$, $x \in \mathbb {R}^d$ with $(\varepsilon ^2+\varepsilon d) \beta < \lambda - L$ that $\mathfrak {u}(x) = u_{\varepsilon }(x)$. This and (82) ensure that for every $v \in \{ w \in C(\mathbb {R}^d,\mathbb {R}) :(\forall \,\varepsilon \in (0,\infty ) :\sup _{x\in \mathbb {R}^d}[(\frac{|w(x)|}{V_{\varepsilon }(x)})] < \infty ) \}$ which satisfies that *v* is a viscosity solution of

84 $$\begin{aligned} \tfrac{1}{2} {\text {Trace}}(BB^{*}({\text {Hess}} v)(x)) = f(x,v(x)) \end{aligned}$$

for $x \in \mathbb {R}^d$ it holds that $v = \mathfrak {u}$. This and (82) establish Item (i). Next note that (83) and the fact that there exist $\varepsilon _n \in (0,\infty )$, $n \in \mathbb {N}$, with $\limsup _{n\rightarrow \infty } \varepsilon _n = 0$ such that $\mathfrak {u} = u_{\varepsilon _n}$ establish Item (ii). The proof of Corollary 2.10 is thus completed. $\square$

### A priori estimates for solutions of SFPEs

In this subsection we establish a priori estimates for solutions of certain SFPEs.

These estimates will be needed as a base case in the MLP approximation analysis in Sect. 3 below.

#### Proposition 2.11

(A priori estimate) Let $d,m \in \mathbb {N}$, $B \in \mathbb {R}^{d\times m}$, $L \in \mathbb {R}$, $\lambda \in ( L, \infty )$, , let $(\Omega ,\mathcal {F},\mathbb {P})$ be a probability space, let $W :[0,\infty ) \times \Omega \rightarrow \mathbb {R}^m$ be a standard Brownian motion, let $f :\mathbb {R}^d\times \mathbb {R}\rightarrow \mathbb {R}$ be measurable, assume for all $x \in \mathbb {R}^d$, $v,w \in \mathbb {R}$ that $|f(x,v)-f(x,w)| \leqslant L | v - w |$ and $\int _0^{\infty } e^{-\lambda t} \, \mathbb {E}[ |f(x+BW_t,0)| ]\,dt < \infty$, let $u :\mathbb {R}^d \rightarrow \mathbb {R}$ be measurable, and assume for all $x \in \mathbb {R}^d$ that $\int _0^{\infty } e^{(\varepsilon -\lambda ) t}\, \mathbb {E}[ |u(x+BW_t)|^2 ]\,dt < \infty$ and

85 $$\begin{aligned} u(x) = \int _0^{\infty } e^{-\lambda t} \, \mathbb {E}\! \left[ f(x+BW_t, u(x+BW_t) ) \right] \!\,dt. \end{aligned}$$

Then

(i) it holds for all $t \in [0,\infty )$, $x \in \mathbb {R}^d$ that
  86 $$\begin{aligned} \begin{aligned}&e^{-\lambda t} \, \mathbb {E}\! \left[ |u(x+BW_t)|^2 \right] \\  &\leqslant \frac{1}{\lambda } \int _t^{\infty } e^{-\lambda s} \, \mathbb {E}\! \left[ |f(x+BW_s,u(x+BW_s))|^2 \right] \!\,ds, \end{aligned} \end{aligned}$$
(ii) it holds for all $x \in \mathbb {R}^d$ that 87 and
(iii) it holds for all $x \in \mathbb {R}^d$ that 88

#### Proof of Proposition 2.11

Throughout this proof let $\nu _{t,x} :\mathcal {B}(\mathbb {R}^d) \rightarrow [0,1]$, $t \in [0,\infty )$, $x \in \mathbb {R}^d$, satisfy for all $t \in [0,\infty )$, $x \in \mathbb {R}^d$, $A \in \mathcal {B}(\mathbb {R}^d)$ that $\nu _{t,x}(A) = \mathbb {P}(x + BW_t \in A)$. Observe that (85) and the Cauchy-Schwarz inequality ensure for all $x \in \mathbb {R}^d$ that

89 $$\begin{aligned} \begin{aligned} |u(x)|^2&\leqslant \frac{1}{\lambda } \int _0^{\infty } e^{-\lambda t} \, \mathbb {E}\! \left[ | f(x+BW_t,u(x+BW_t)) |^2 \right] \!\,dt. \end{aligned} \end{aligned}$$

This, Fubini’s theorem, and the fact that for all $t, s\in [0,\infty )$, $x \in \mathbb {R}^d$, $A \in \mathcal {B}(\mathbb {R}^d)$ it holds that $\nu _{t+s,x}(A) = \int _{\mathbb {R}^d} \nu _{s,y}(A)\,\nu _{t,x}(dy)$ ensure for all $t \in [0,\infty )$ $x \in \mathbb {R}^d$ that

90 $$\begin{aligned} \begin{aligned} \mathbb {E}\! \left[ |u(x+BW_t)|^2 \right]&= \int _{\mathbb {R}^d} |u(y)|^2\,\nu _{t,x}(dy) \\&\leqslant \frac{1}{\lambda } \int _{\mathbb {R}^d} \int _0^{\infty } e^{-\lambda s}\, \mathbb {E}\! \left[ |f(y+BW_{s},u(y+BW_s))|^2 \right] \!\,ds \,\nu _{t,x}(dy) \\&= \frac{1}{\lambda } \int _0^{\infty } e^{-\lambda s} \, \mathbb {E}\! \left[ |f(x+BW_{t+s},u(x+BW_{t+s}))|^2 \right] \!\,ds \\&= \frac{e^{\lambda t}}{\lambda } \int _t^{\infty } e^{-\lambda s} \, \mathbb {E}\! \left[ |f(x+BW_{s},u(x+BW_{s}))|^2 \right] \!\,ds. \end{aligned} \end{aligned}$$

This establishes Item (i). Combining Item (i) with Fubini’s theorem ensures for all $x \in \mathbb {R}^d$ that

91 $$\begin{aligned} \begin{aligned}&\int _0^{\infty } e^{(\varepsilon -\lambda )t} \, \mathbb {E}\! \left[ |u(x+BW_t)|^2 \right] \!\,dt \\&\leqslant \int _0^{\infty } \frac{e^{\varepsilon t} }{\lambda } \, \int _t^{\infty } e^{-\lambda s} \, \mathbb {E}\! \left[ | f(x+BW_s,u(x+BW_s))|^2 \right] \!\,ds \,dt \\&= \int _0^{\infty } \left[ \int _0^s \frac{e^{\varepsilon t} }{\lambda } \,dt \right] e^{-\lambda s} \, \mathbb {E}\! \left[ |f(x+BW_s,u(x+BW_s))|^2 \right] \!\,ds \\&\leqslant \frac{1}{\varepsilon \lambda } \int _0^{\infty } e^{(\varepsilon -\lambda )s} \, \mathbb {E}\! \left[ |f(x+BW_s,u(x+BW_s))|^2 \right] \!\,ds. \end{aligned} \end{aligned}$$

Minkowski’s inequality hence shows for all $x \in \mathbb {R}^d$ that9293This implies for all $x \in \mathbb {R}^d$ that94This establishes Item (ii). Next observe that the triangle inequality, Fubini’s theorem, the assumption that for all $x \in \mathbb {R}^d$, $a,b \in \mathbb {R}$ it holds that $| f(x,a) - f(x,b) | \leqslant L |a-b|$, and (85) ensure for all $x \in \mathbb {R}^d$ that

95 $$\begin{aligned} | u(x) | \leqslant \int _0^{\infty } e^{-\lambda t } \, \mathbb {E}\! \left[ | f(x+BW_t,0) | \right] \!\,dt + L \int _0^{\infty } e^{-\lambda t} \, \mathbb {E}\! \left[ |u(x+BW_t)| \right] \!\,dt. \end{aligned}$$

The Cauchy-Schwarz inequality and Item (ii) hence prove for all $x \in \mathbb {R}^d$ that96This establishes Item (iii). The proof of Proposition 2.11 is thus completed. $\square$

## Full-history recursive multilevel Picard (MLP) approximations

In this section we introduce and analyze MLP approximation schemes for SFPEs related to semilinear elliptic PDEs (see Setting 3.1 in Sect. 3.1 below). In Sects. 3.2-3.3 we establish some rather technical results concerning measurability, distributional, and integrability properties of MLP approximations. Section 3.4 contains fundamental estimates for the biases and variances of MLP approximations (see Lemmas 3.6 and 3.7 below). These fundamental estimates for the biases and variances of MLP approximations are merged in Proposition 3.9 and Corollary3.10 in Sect. 3.5 below to obtain a full error analysis for MLP approximations in Corollary 3.11 in Sect. 3.5 below. In Sect. 3.6 we provide an upper bound for the computational cost to sample realizations of MLP approximations. In Sect. 3.7 we finally relate the error analysis for MLP approximations established in Corollary 3.11 in Sect. 3.5 with the computational cost analysis for MLP approximations established in Sect. 3.6 to obtain in Theorem 3.16 in Sect. 3.7 an overall complexity analysis for the proposed MLP approximation schemes.

### MLP approximations

In this subsection we introduce MLP approximation schemes for SFPEs related to semilinear elliptic PDEs.

#### Setting 3.1

Let $d, M \in \mathbb {N}$, $\lambda \in (0,\infty )$, $\Theta = \cup _{n\in \mathbb {N}} \mathbb {Z}^n$, $B \in \mathbb {R}^{d\times d}$, $f \in C( \mathbb {R}^d \times \mathbb {R}, \mathbb {R})$, let $( \Omega , \mathcal {F}, \mathbb {P})$ be a probability space, let $W^{\theta } :[0,\infty ) \times \Omega \rightarrow \mathbb {R}^d$, $\theta \in \Theta$, be i.i.d. standard Brownian motions, let $R^{\theta }:\Omega \rightarrow [0,\infty )$, $\theta \in \Theta$, be i.i.d. random variables which satisfy for all $t\in [0,\infty )$ that $\mathbb {P}(R^{0}\geqslant t)=e^{-\lambda t}$, assume that $(R^{\theta })_{\theta \in \Theta }$ and $(W^{\theta })_{\theta \in \Theta }$ are independent, and let $U^{\theta }_{n} = ( U^{\theta }_n (x) )_{ x \in \mathbb {R}^d } :\mathbb {R}^d \times \Omega \rightarrow \mathbb {R}$, $\theta \in \Theta$, $n \in \mathbb {N}_0$, satisfy for all $\theta \in \Theta$, $n \in \mathbb {N}$, $x \in \mathbb {R}^d$ that $U^{\theta }_{0}(x)=0$ and

97 $$\begin{aligned} \begin{aligned}&U^{\theta }_{n}(x) \\  &= \sum _{k=1}^{n-1} \frac{1}{\lambda M^{n-k}} \Bigg [ \sum _{m=1}^{M^{n-k}} \Big ( f\big (x+BW^{(\theta ,k,m)}_{R^{(\theta ,k,m)}},U^{(\theta ,k,m)}_{k}( x+BW^{(\theta ,k,m)}_{R^{(\theta ,k,m)}})\big ) \\&- f\big (x+BW^{(\theta ,k,m)}_{R^{(\theta ,k,m)}},U^{(\theta ,k,-m)}_{k-1}(x+BW^{(\theta ,k,m)}_{R^{(\theta ,k,m)}})\big ) \Big ) \Bigg ] \\  &+ \frac{1}{\lambda M^n}\left[ \sum _{m=1}^{M^n} f\big (x+BW^{(\theta ,0,m)}_{R^{(\theta ,0,m)}},0\big ) \right] \!. \end{aligned} \end{aligned}$$

### Measurability properties of MLP approximations

In this subsection we establish elementary measurability properties of MLP approximations. A similar result for related semilinear parabolic PDEs can be found in [47, Lemma 3.2].

#### Lemma 3.2

Assume Setting 3.1. Then

(i) it holds for all $n \in \mathbb {N}_0$, $\theta \in \Theta$ that $U^{\theta }_n :\mathbb {R}^d \times \Omega \rightarrow \mathbb {R}$ is a continuous random field,
(ii) it holds for all $n \in \mathbb {N}_0$, $\theta \in \Theta$ that $\sigma _{\Omega }(U^{\theta }_n) \subseteq \sigma _{\Omega }((R^{(\theta ,\vartheta )})_{\vartheta \in \Theta },(W^{(\theta ,\vartheta )})_{\vartheta \in \Theta })$,
(iii) it holds that $\mathbb {R}^d\times \Omega \ni (x,\omega ) \mapsto x+BW^{\theta }_{R^{\theta }(\omega )}(\omega ) \in \mathbb {R}^d$, $\theta \in \Theta$, are identically distributed random fields, and
(iv) it holds for all $n\in \mathbb {N}_0$ that $U^{\theta }_n$, $\theta \in \Theta$, are identically distributed random fields.

#### Proof of Lemma 3.2

Lemma 3.2 is an immediate consequence of (97) and [47, Corollary 2.5]. A detailed proof can be found, e.g., in the extended preprint version [7, Lemma 3.2] of this article. $\square$

### Integrability properties of MLP approximations

In this subsection we establish basic integrability properties of MLP approximations.

#### Lemma 3.3

Assume Setting 3.1, let $p \in [1,\infty )$, $k \in \mathbb {N}_0$, $\theta ,\vartheta \in \Theta$, assume that $\theta \notin \{(\vartheta ,\eta ):\eta \in \Theta \}$, and let $\nu _{t,x} :\mathcal {B}(\mathbb {R}^d) \rightarrow [0,\infty )$, $t\in [0,\infty )$, $x \in \mathbb {R}^d$, satisfy for all $t \in [0,\infty )$, $x \in \mathbb {R}^d$, $A \in \mathcal {B}(\mathbb {R}^d)$ that $\nu _{t,x}(A) = \mathbb {P}( x + BW^0_t \in A )$. Then

(i) it holds for all $x \in \mathbb {R}^d$ that
  98 $$\begin{aligned} \begin{aligned} \mathbb {E}\! \left[ | U^{\vartheta }_k(x + BW^{\theta }_{R^{\theta }}) |^p \right]&= \int _0^{\infty } \lambda e^{-\lambda s} \, \mathbb {E}\! \left[ |U^{\vartheta }_k(x+BW^{\theta }_s)|^p \right] \!\,ds \end{aligned} \end{aligned}$$
  and
(ii) it holds for all $x \in \mathbb {R}^d$, $t \in [0,\infty )$ that
  99 $$\begin{aligned} \mathbb {E}\! \left[ |U^{\vartheta }_k(x+BW^{\theta }_t)|^p \right] = \int _{\mathbb {R}^d} \mathbb {E}\! \left[ |U^0_k(y)|^p \right] \!\, \nu _{t,x}(dy). \end{aligned}$$

#### Proof of Lemma 3.3

Lemma 3.3 is an immediate consequence of Item (ii) in Lemma 3.2, the assumption that $\theta \notin \{(\vartheta ,\eta ):\eta \in \Theta \}$, [47, Lemma 2.2], the assumption that for all $t \in (0,\infty )$ it holds that $\mathbb {P}(R^{\theta } \geqslant t) = e^{-\lambda t}$, and Item (iv) in Lemma 3.2. A detailed proof can be found, e.g., in the extended preprint version [7, Lemma 3.3] of this article. $\square$

The next result shows that in the considered setting certain integrability properties of the function *f* imply analogous integrability properties for the MLP approximations.

#### Lemma 3.4

Assume Setting 3.1, let $\varepsilon \in (0,\infty )$, $p \in [1,\infty )$, $L \in \mathbb {R}$, and assume for all $x \in \mathbb {R}^d$, $v,w \in \mathbb {R}$ that $| f ( x, v ) - f ( x, w ) | \leqslant L | v - w |$ and $\int _0^{\infty } e^{(\varepsilon -\lambda ) s}\, \mathbb {E}[ | f ( x+BW^{0}_s, 0 ) |^p ] \,ds<\infty$. Then it holds for all $n \in \mathbb {N}_0$, $x \in \mathbb {R}^d$ that

100 $$\begin{aligned} \int _0^{\infty } e^{(\varepsilon -\lambda ) s} \, \mathbb {E}\! \left[ | U^{0}_n(x+BW^{0}_{s}) |^p \right] \!\,ds < \infty . \end{aligned}$$

#### Proof of Lemma 3.4

Throughout this proof let $x \in \mathbb {R}^d$, let $\mathbb {A}\subseteq \mathbb {N}$ be the set given by

101 $$\begin{aligned} \mathbb {A}= \left\{ n \in \mathbb {N}:\left( \forall \,k \in \{0,1,\ldots ,n-1\}:\int _0^{\infty } e^{(\varepsilon -\lambda ) s}\, \mathbb {E}\! \left[ |U^0_k(x+BW^{0}_s)|^p \right] \!\,ds < \infty \right) \right\} \!, \end{aligned}$$

and let $\nu _{t,y} :\mathcal {B}(\mathbb {R}^d) \rightarrow [0,\infty )$, $t\in [0,\infty )$, $y \in \mathbb {R}^d$, satisfy for all $t \in [0,\infty )$, $y \in \mathbb {R}^d$, $A \in \mathcal {B}(\mathbb {R}^d)$ that $\nu _{t,y}(A) = \mathbb {P}( y + BW^0_t \in A)$. Observe that the assumption that for all $y \in \mathbb {R}^d$ it holds that $U^0_0(y) = 0$ ensures that $1 \in \mathbb {A}$. Moreover, note that (97), the fact that for all $a,b \in \mathbb {R}$ it holds that $|a+b|^p \leqslant 2^{p-1} |a|^p + 2^{p-1} |b|^p$, and the assumption that for all $y \in \mathbb {R}^d$, $v,w \in \mathbb {R}$ it holds that $| f(y,v) - f(y,w) | \leqslant L | v-w |$ ensure for all $n \in \mathbb {N}$, $y \in \mathbb {R}^d$ that

102 $$\begin{aligned} \begin{aligned} |U^0_n(y)|^p&\leqslant 2^{p-1} \left[ \sum _{k=1}^{n-1} \frac{1}{\lambda M^{n-k}} \sum _{m=1}^{M^{n-k}} L \biggl | U^{(0,k,m)}_k(y+BW^{(0,k,m)}_{R^{(0,k,m)}})\right. \\  &\left. - U^{(0,k,-m)}_{k-1}(y+BW^{(0,k,m)}_{R^{(0,k,m)}}) \biggr | \right] ^p \\&+ 2^{p-1} \left[ \frac{1}{\lambda M^n} \sum _{m=1}^{M^n} \left| f(y+BW^{(0,0,m)}_{R^{(0,0,m)}},0) \right| \right] ^p. \end{aligned} \end{aligned}$$

This and the fact that $[0,\infty ) \ni t \mapsto t^p \in [0,\infty )$ is convex guarantee for all $n \in \mathbb {N}$, $y \in \mathbb {R}^d$ that

103 $$\begin{aligned} \begin{aligned}&|U^0_n(y)|^p \\  &\leqslant \frac{2^{p-1}}{\lambda ^p M^n} \sum _{m=1}^{M^n} \left| f(y+BW^{(0,0,m)}_{R^{(0,0,m)}},0) \right| ^p \\&+ \sum _{k=1}^{n-1} \frac{2^{p-1} n^p L^p}{\lambda ^p M^{n-k}} \sum _{m=1}^{M^{n-k}} \left| U^{(0,k,m)}_{k}(y+BW^{(0,k,m)}_{R^{(0,k,m)}}) - U^{(0,k,-m)}_{k-1}(y+BW^{(0,k,m)}_{R^{(0,k,m)}}) \right| ^p \\&\leqslant \frac{2^{p-1}}{\lambda ^p M^n} \sum _{m=1}^{M^n} \left| f(y+BW^{(0,0,m)}_{R^{(0,0,m)}},0) \right| ^p \\&+ \sum _{k=1}^{n-1} \frac{4^{p-1}n^pL^p}{\lambda ^p M^{n-k}} \sum _{m=1}^{M^{n-k}} \biggl [ \Bigl | U^{(0,k,m)}_{k}(y+BW^{(0,k,m)}_{R^{(0,k,m)}}) \Bigr |^p \\  &+ \Bigl | U^{(0,k,-m)}_{k-1}(y+BW^{(0,k,m)}_{R^{(0,k,m)}}) \Bigr |^p \biggr ]. \end{aligned} \end{aligned}$$

Item (iii) in Lemma 3.2 and Item (i) in Lemma 3.3 hence ensure for all $n \in \mathbb {N}$, $y \in \mathbb {R}^d$ that

104 $$\begin{aligned}&\mathbb {E}\! \left[ |U^0_n(y)|^p \right] \leqslant \frac{2^{p-1}}{\lambda ^{p-1}} \int _0^{\infty } e^{-\lambda s} \, \mathbb {E}\! \left[ \left| f(y+BW^{0}_{s},0) \right| ^p \right] \!\,ds \end{aligned}$$

105 $$\begin{aligned}&+ \sum _{k=1}^{n-1} \frac{4^{p-1}n^pL^p}{\lambda ^{p-1} M^{n-k}} \sum _{m=1}^{M^{n-k}} \int _0^{\infty } e^{-\lambda s}\, \mathbb {E}\biggl [ \Bigl | U^{(0,k,m)}_{k}(y+BW^{(0,k,m)}_{s}) \Bigr |^p\nonumber \\&+ \Bigl | U^{(0,k,-m)}_{k-1}(y+BW^{(0,k,m)}_s) \Bigr |^p \biggr ] \, ds. \end{aligned}$$

Item (ii) in Lemma 3.3 hence guarantees for all $n \in \mathbb {N}$, $y \in \mathbb {R}^d$ that

106 $$\begin{aligned} \begin{aligned}&\mathbb {E}\! \left[ |U^0_n(y)|^p \right] \leqslant \frac{2^{p-1}}{\lambda ^{p-1}} \int _0^{\infty } e^{-\lambda s} \, \mathbb {E}\! \left[ \left| f(y+BW^{0}_{s},0) \right| ^p \right] \!\,ds \\&+ \sum _{k=1}^{n-1} \frac{4^{p-1}n^pL^p }{\lambda ^{p-1}} \int _0^{\infty } e^{-\lambda s}\, \mathbb {E}\! \left[ \left| U^{0}_{k}(y+BW^{0}_{s})\right| ^p + \left| U^{0}_{k-1}(y+BW^{0}_{s}) \right| ^p \right] \!\,ds. \\&\leqslant \frac{2^{p-1}}{\lambda ^{p-1}} \int _0^{\infty } e^{-\lambda s} \left[ \int _{\mathbb {R}^d} \left( \left| f(z,0) \right| ^p + 2^pn^pL^p \sum _{k=0}^{n-1} \mathbb {E}\! \left[ \left| U^{0}_{k}(z)\right| ^p \right] \!\right) \!\, \nu _{s,y}(dz) \right] \!\,ds. \end{aligned} \end{aligned}$$

Item (ii) in Lemma 3.3, Fubini’s theorem, and the fact that for all $t,s\in [0,\infty )$, $A \in \mathcal {B}(\mathbb {R}^d)$ it holds that $\nu _{t+s,x}(A) = \int _{\mathbb {R}^d} \nu _{s,y}(A) \, \nu _{t,x}(dy)$ therefore ensure for all $n \in \mathbb {N}$, $t \in [0, \infty )$ that

107 $$\begin{aligned}&\mathbb {E}\! \left[ |U^0_n(x+BW^{0}_t)|^p \right] = \int _{\mathbb {R}^d} \mathbb {E}\! \left[ |U^0_n(y)|^p \right] \!\,\nu _{t,x}(dy) \nonumber \\&\leqslant \frac{2^{p-1}}{\lambda ^{p-1}} \int _{\mathbb {R}^d} \int _0^{\infty } e^{-\lambda s} \Biggl [ \int _{\mathbb {R}^d} \biggl ( \left| f(z,0) \right| ^p \nonumber \\  &+ 2^pn^pL^p \sum _{k=0}^{n-1} \mathbb {E}\! \left[ \left| U^{0}_{k}(z)\right| ^p \right] \biggr ) \nu _{s,y}(dz) \Biggr ] \, ds \, \nu _{t,x}(dy) \nonumber \\&= \frac{2^{p-1}}{\lambda ^{p-1}} \int _0^{\infty } e^{-\lambda s} \left[ \int _{\mathbb {R}^d} \left( \left| f(z,0) \right| ^p + 2^pn^pL^p \sum _{k=0}^{n-1} \mathbb {E}\! \left[ \left| U^{0}_{k}(z)\right| ^p \right] \!\right) \!\, \nu _{t+s,x}(dz) \right] \!\,ds. \end{aligned}$$

Combining this with Item (ii) in Lemma 3.3 demonstrates for all $n \in \mathbb {N}$, $t \in [0,\infty )$ that

108 $$\begin{aligned} \begin{aligned}&\mathbb {E}\! \left[ |U^0_n(x+BW^{0}_t)|^p \right] \\&\leqslant \frac{2^{p-1}}{\lambda ^{p-1}} \int _0^{\infty } e^{-\lambda s} \Biggl [ \mathbb {E}\! \left[ |f(x+BW^{0}_{t+s},0)|^p \right] \\  &+ 2^pn^pL^p \sum _{k=0}^{n-1} \mathbb {E}\! \left[ |U^0_k(x+BW^{0}_{t+s})|^p \right] \Biggr ] \, ds \\&= e^{\lambda t} \frac{2^{p-1}}{\lambda ^{p-1}} \int _t^{\infty } e^{-\lambda s} \Biggl ( \mathbb {E}\! \left[ |f(x+BW^{0}_s,0)|^p \right] \\  &+ 2^pn^pL^p \sum _{k=0}^{n-1} \mathbb {E}\! \left[ |U^0_k(x+BW^{0}_{s})|^p \right] \Biggr ) \, ds. \end{aligned} \end{aligned}$$

Hence, we obtain for all $n \in \mathbb {N}$ that

109 $$\begin{aligned}&\int _0^{\infty } e^{(\varepsilon -\lambda ) t} \, \mathbb {E}\! \left[ |U^{0}_n(x+BW^{0}_{t}) |^p \right] \!\,dt \nonumber \\&\leqslant \frac{2^{p-1}}{\lambda ^{p-1}} \int _0^{\infty } e^{\varepsilon t} \int _t^{\infty } e^{-\lambda s} \Biggl ( \mathbb {E}\! \left[ |f(x+BW^{0}_s,0)|^p \right] \nonumber \\  &+ 2^pn^pL^p \sum _{k=0}^{n-1} \mathbb {E}\! \left[ |U^0_k(x+BW^{0}_{s})|^p \right] \Biggr ) \, ds \, dt \nonumber \\&= \frac{2^{p-1}}{\lambda ^{p-1}} \int _0^{\infty } e^{-\lambda s} \Biggl ( \mathbb {E}\! \left[ |f(x+BW^{0}_s,0)|^p \right] \nonumber \\  &+ 2^pn^pL^p \sum _{k=0}^{n-1} \mathbb {E}\! \left[ |U^0_k(x+BW^{0}_{s})|^p \right] \Biggr ) \left[ \int _0^s e^{\varepsilon t} \,dt \right] \!\,ds \nonumber \\&\leqslant \frac{2^{p-1}}{\lambda ^{p-1}\varepsilon } \int _0^{\infty } e^{(\varepsilon -\lambda ) s} \Biggl ( \mathbb {E}\! \left[ |f(x+BW^{0}_s,0)|^p \right] \nonumber \\  &+ 2^pn^pL^p \sum _{k=0}^{n-1} \mathbb {E}\! \left[ |U^0_k(x+BW^{0}_{s})|^p \right] \Biggr )\,ds. \end{aligned}$$

This implies for all $n \in \mathbb {A}$ that $\int _0^{\infty } e^{(\varepsilon -\lambda )s} \, \mathbb {E}[ |U^0_n(x+BW^{0}_s)|^p ]\,ds < \infty$. Therefore, we obtain for all $n \in \mathbb {N}$ that $(n \in \mathbb {A}\Rightarrow n+1 \in \mathbb {A})$. Combining this with the fact that $1 \in \mathbb {A}$ and induction demonstrates that $\mathbb {N}\subseteq \mathbb {A}$. This establishes (100). The proof of Lemma 3.4 is thus completed. $\square$

### Bias and variance estimates for MLP approximations

In this subsection we establish fundamental estimates for the biases and variances of MLP approximations, see Lemmas 3.6 and 3.7 below.

In Lemma 3.5 below we establish an elementary result stating that the expected values of MLP approximations satisfy certain relations similar to the SFPE satisfied by viscosity solutions of certain semilinear elliptic PDEs as considered in Sect. 2. Lemma 3.5 will allow us to estimate the bias of MLP approximations by average approximation errors of lower-level MLP approximations, see Lemma 3.6 below.

#### Lemma 3.5

(Mean) Assume Setting 3.1, let $L\in \mathbb {R}$, $\varepsilon \in (0,\infty )$, and assume for all $x\in \mathbb {R}^d$, $v,w\in \mathbb {R}$ that $|f(x,v)-f(x,w)|\leqslant L|v-w|$ and $\int _0^{\infty } e^{(\varepsilon -\lambda ) s}\, \mathbb {E}[ |f(x+BW^{0}_s,0)| ]\,ds<\infty$. Then

(i) it holds for all $n \in \mathbb {N}$, $x \in \mathbb {R}^d$ that $\int _0^{\infty } e^{(\varepsilon -\lambda ) s}\, \mathbb {E}[ |f(x+BW^{0}_s,U^{0}_{n-1}(x+BW^{0}_s))| ]\,ds<\infty$ and
(ii) it holds for all $n \in \mathbb {N}$, $\theta \in \Theta$, $x \in \mathbb {R}^d$ that
  110 $$\begin{aligned} \mathbb {E}\! \left[ U^{\theta }_{n}(x) \right] = \tfrac{1}{\lambda } \mathbb {E}\! \left[ f\big (x+BW^{0}_{R^0},U^{0}_{n-1}(x+BW^{0}_{R^{0}})\big ) \right] . \end{aligned}$$

#### Proof of Lemma 3.5

First, note that Lemma 3.4, the assumption that for all $x \in \mathbb {R}^d$ it holds that $\int _0^{\infty } e^{(\varepsilon -\lambda ) s}\, \mathbb {E}[ |f(x+BW^{0}_s,0)| ]\,ds < \infty$, and the assumption that for all $x \in \mathbb {R}^d$, $v,w \in \mathbb {R}$ it holds that $| f(x,v) - f(x,w) | \leqslant L |v-w|$ ensure for all $n \in \mathbb {N}$, $x \in \mathbb {R}^d$ that

111 $$\begin{aligned} \begin{aligned}&\int _0^{\infty } e^{(\varepsilon -\lambda )t}\, \mathbb {E}\! \left[ |f(x+BW^{0}_t,U^0_{n-1}(x+BW^{0}_t))| \right] \!\,dt \\&\leqslant \int _0^{\infty } e^{(\varepsilon -\lambda )t}\, \mathbb {E}\! \left[ |f(x+BW^{0}_t,0)| \right] \!\,dt + L \int _0^{\infty } e^{(\varepsilon -\lambda )t}\, \mathbb {E}\! \left[ |U^0_{n-1}(x+BW^{0}_t)| \right] \!\,dt < \infty . \end{aligned} \end{aligned}$$

This establishes Item (i). Next note that Item (i), Items (i)–(iv) in Lemma 3.2, and [47, Corollary 2.5] ensure for all $\theta \in \Theta$, $n \in \mathbb {N}$, $x \in \mathbb {R}^d$ that

$$\begin{aligned} \begin{aligned}&\mathbb {E}\! \left[ U^{\theta }_{n}(x) \right] \\&= \sum _{k=1}^{n-1} \frac{1}{\lambda M^{n-k}} \sum _{m=1}^{M^{n-k}} \bigg ( \mathbb {E}\! \left[ f\Big (x+BW^{(\theta ,k,m)}_{R^{(\theta ,k,m)}},U^{(\theta ,k,m)}_{k}\big (x+BW^{(\theta ,k,m)}_{R^{(\theta ,k,m)}}\big )\Big ) \right] \\&- \mathbb {E}\! \left[ f\Big ( x+BW^{(\theta ,k,m)}_{R^{(\theta ,k,m)}},U^{(\theta ,k,-m)}_{k-1}\big (x+BW^{(\theta ,k,m)}_{R^{(\theta ,k,m)}}\big ) \Big ) \right] \bigg ) \\&+ \frac{1}{\lambda M^n} \sum _{m=1}^{M^{n}} \mathbb {E}\! \left[ f\big (x+BW^{(\theta ,0,m)}_{R^{(\theta ,0,m)}},0\big ) \right] \! \\&= \sum _{k=1}^{n-1} \frac{1}{\lambda } \bigg ( \mathbb {E}\! \left[ f\big (x+BW^{0}_{R^{0}},U^{0}_{k}(x+BW^{0}_{R^{0}})\big ) \right] \\  &- \mathbb {E}\! \left[ f\big (x+BW^{0}_{R^{0}},U^{0}_{k-1}(x+BW^{0}_{R^{0}})\big ) \right] \bigg ) + \frac{1}{\lambda } \mathbb {E}\! \left[ f\big (x+BW^{0}_{R^{0}},0\big ) \right] . \end{aligned} \end{aligned}$$

The assumption that for all $x \in \mathbb {R}^d$ it holds that $U^0_0(x) = 0$ hence proves for all $\theta \in \Theta$, $n \in \mathbb {N}$, $x \in \mathbb {R}^d$ that

112 $$\begin{aligned} \begin{aligned} \mathbb {E}\! \left[ U^{\theta }_{n}(x) \right]&= \frac{1}{\lambda } \Big ( \mathbb {E}\! \left[ f\big (x+BW^0_{R^{0}},0\big ) \right] \\&+ \mathbb {E}\! \left[ f\big (x+BW^0_{R^{0}},U^{0}_{n-1}(x+BW^0_{R^{0}})\big ) \right] \\  &- \mathbb {E}\! \left[ f\big (x+BW^0_{R^{0}},U^{0}_{0}(x+BW^0_{R^{0}})\big ) \right] \Big ) \\&= \frac{1}{\lambda } \mathbb {E}\! \left[ f\big (x+BW^{0}_{R^0},U^{0}_{n-1}(x+BW^{0}_{R^{0}})\big ) \right] \!. \end{aligned} \end{aligned}$$

This establishes Item (ii). The proof of Lemma 3.5 is thus completed. $\square$

In the next result we combine Lemma 3.5 above with a Lipschitz continuity assumption to estimate the bias of MLP approximations.

#### Lemma 3.6

(Bias) Assume Setting 3.1, let $L \in \mathbb {R}$, $\varepsilon \in (0,\infty )$, $p \in [1,\infty )$, assume for all $x \in \mathbb {R}^d$, $v, w \in \mathbb {R}$ that $| f ( x, v ) - f ( x, w ) | \leqslant L | v - w |$ and $\int _0^{\infty } e^{(\varepsilon -\lambda ) s} \, \mathbb {E}[ |f(x+BW^{0}_s,0)| ]\,ds<\infty$, let $u :\mathbb {R}^d \rightarrow \mathbb {R}$ be measurable, and assume for all $x \in \mathbb {R}^d$ that $\int _0^{\infty } e^{ -\lambda s } \, \mathbb {E}[ | u ( x+BW^{ 0 }_s ) | ]\,ds < \infty$ and

113 $$\begin{aligned} \begin{aligned} u(x)&= \frac{1}{\lambda } \mathbb {E}\! \left[ f(x+BW^{0}_{R^0},u(x+BW^{0}_{R^0})) \right] \\  &= \mathbb {E}\! \left[ \int _0^{\infty } e^{-\lambda s} f(x+BW^{0}_s,u(x+BW^{0}_s))\,ds \right] \!. \end{aligned} \end{aligned}$$

Then it holds for all $n \in \mathbb {N}$, $\theta \in \Theta$, $x \in \mathbb {R}^d$ that

114 $$\begin{aligned} \left| \mathbb {E}\! \left[ U^{\theta }_{n}(x) \right] -u(x)\right| ^p \leqslant \frac{L^p}{\lambda ^p} \mathbb {E}\! \left[ |U^0_{n-1}(x+BW^{0}_{R^{0}})-u(x+BW^{0}_{R^0})|^p \right] \!. \end{aligned}$$

#### Proof of Lemma 3.6

First, observe that Lemma 3.5 and (113) guarantee for all $n \in \mathbb {N}$, $\theta \in \Theta$, $x \in \mathbb {R}^d$ that

115 $$\begin{aligned} \begin{aligned}&\mathbb {E}\! \left[ U^{\theta }_{n}(x) \right] -u(x) \\  &= \frac{1}{\lambda } \mathbb {E}\! \left[ f(x+BW^{0}_{R^0},U^0_{n-1}(x+BW^{0}_{R^0})) - f(x+BW^{0}_{R^0},u(x+BW^{0}_{R^0})) \right] \!. \end{aligned} \end{aligned}$$

The assumption that for all $x \in \mathbb {R}^d$, $v,w \in \mathbb {R}$ it holds that $|f(x,v)-f(x,w)|\leqslant L|v-w|$ therefore implies for all $n \in \mathbb {N}$, $\theta \in \Theta$, $x \in \mathbb {R}^d$ that

116 $$\begin{aligned} \left| \mathbb {E}\! \left[ U^{\theta }_{n}(x) \right] -u(x)\right| \leqslant \frac{L}{\lambda } \mathbb {E}\! \left[ |U^0_{n-1}(x+BW^{0}_{R^0})-u(x+BW^{0}_{R^0})| \right] \!. \end{aligned}$$

Jensen’s inequality hence establishes (114). The proof of Lemma 3.6 is thus completed. $\square$

In Lemma 3.7 below we estimate the variance of MLP approximations. Our proof combines a result from [47] with Lemma 3.2 and Lemma 3.5 above.

#### Lemma 3.7

(Variance) Assume Setting 3.1, let $L \in \mathbb {R}$, $\varepsilon \in (0,\infty )$, and assume for all $x \in \mathbb {R}^d$, $v,w \in \mathbb {R}$ that $|f(x,v)-f(x,w)|\leqslant L|v-w|$ and $\int _0^{\infty } e^{(\varepsilon -\lambda ) s}\, \mathbb {E}[ |f(x+BW^0_s,0)| ]\,ds<\infty$. Then it holds for all $n \in \mathbb {N}$, $\theta \in \Theta$, $x \in \mathbb {R}^d$ that

117 $$\begin{aligned} \begin{aligned}&{\text {Var}} [ U^{\theta }_{n}(x) ] \\&\leqslant \frac{1}{\lambda ^2} \bigg [ \frac{1}{M^n} \mathbb {E}\! \left[ |f(x+BW^{0}_{R^0},0)|^2 \right] \\  &+ \sum _{k=1}^{n-1} \frac{L^2}{M^{n-k}} \mathbb {E}\! \left[ |U^0_{k}(x+BW^{0}_{R^0})-U^1_{k-1}(x+BW^{0}_{R^0})|^2 \right] \bigg ]. \end{aligned} \end{aligned}$$

#### Proof of Lemma 3.7

First, observe that Item (ii) in Lemma 3.2 and Setting 3.1 ensure for all $\theta \in \Theta$, $x \in \mathbb {R}^d$ that

118 $$\begin{aligned} \begin{aligned}&\mathbb {N}_0 \times \mathbb {N}\ni (k,m) \mapsto \\&{\left\{ \begin{array}{ll} \left( \begin{array}{l} \Omega \ni \omega \mapsto f(x+BW^{(\theta ,k,m)}_{R^{(\theta ,k,m)}(\omega )}(\omega ), \\ U_k^{(\theta ,k,m)}(x+BW^{(\theta ,k,m)}_{R^{(\theta ,k,m)}(\omega )}(\omega ),\omega )) \\ - f(x+BW^{(\theta ,k,m)}_{R^{(\theta ,k,m)}(\omega )}(\omega ), \\ U_{k-1}^{(\theta ,k,-m)}(x+BW^{(\theta ,k,m)}_{R^{(\theta ,k,m)}(\omega )}(\omega ),\omega )) \in \mathbb {R}\end{array} \right) &  :k\geqslant 1 \\ \Omega \ni \omega \mapsto f(x+BW^{(\theta ,0,m)}_{R^{(\theta ,0,m)}(\omega )}(\omega ),0) \in \mathbb {R}&  :k=0 \end{array}\right. } \end{aligned} \end{aligned}$$

is an independent family of random variables. This, Item (i) in Lemma 3.5, and (97) imply for all $n \in \mathbb {N}$, $\theta \in \Theta$, $x \in \mathbb {R}^d$ that

119 $$\begin{aligned} \begin{aligned}&{\text {Var}} [ U^{\theta }_{n}(x) ] \\  &= \frac{1}{\lambda ^2} \Bigg ( \sum _{m=1}^{M^{n}} {\text {Var}}\!\left[ \frac{f(x+BW^{(\theta ,0,m)}_{R^{(\theta ,0,m)}},0)}{M^n} \right] \\&+ \sum _{k=1}^{n-1} \sum _{m=1}^{M^{n-k}} {\text {Var}} \Bigg [ \frac{ f(x+BW^{(\theta ,k,m)}_{R^{(\theta ,k,m)}},U^{(\theta ,k,m)}_{k}(x+BW^{(\theta ,k,m)}_{R^{(\theta ,k,m)}}) ) }{M^{n-k}} \\&- \frac{f(x+BW^{(\theta ,k,m)}_{R^{(\theta ,k,m)}},U^{(\theta ,k,-m)}_{k-1}(x+BW^{(\theta ,k,m)}_{R^{(\theta ,k,m)}})) }{M^{n-k}} \Bigg ] \Bigg ). \end{aligned} \end{aligned}$$

Items (i)–(iv) in Lemma 3.2 and [47, Corollary 2.5], the fact that for every random variable $Y:\Omega \rightarrow \mathbb {R}$ with $\mathbb {E}\! \left[ |Y| \right] <\infty$ it holds that ${\text {Var}} [ Y ] \leqslant \mathbb {E}\! \left[ Y^2 \right] \in [0,\infty ]$, and the assumption that for all $x \in \mathbb {R}^d$, $v,w \in \mathbb {R}$ it holds that $|f(x,v)-f(x,w)| \leqslant L |v-w|$ therefore imply for all $n \in \mathbb {N}$, $\theta \in \Theta$, $x \in \mathbb {R}^d$ that

120 $$\begin{aligned} \begin{aligned}&{\text {Var}}\!\left[ U^{\theta }_n(x) \right] = \frac{1}{\lambda ^2} \Bigg [ \frac{1}{M^n} {\text {Var}} [ f(x+BW^{0}_{R^{0}},0) ] \\&+ \sum _{k=1}^{n-1} \frac{1}{M^{n-k}} {\text {Var}} \Big [ f(x+BW^{0}_{R^{0}},U^{0}_{k}(x+BW^{0}_{R^{0}} ) ) \\  &- f(x+BW^{0}_{R^{0}},U^{1}_{k-1}(x+BW^{0}_{R^{0}})) \Big ] \Bigg ] \\&\leqslant \frac{1}{\lambda ^2} \bigg [ \frac{1}{M^n} \mathbb {E}\! \left[ |f(x+BW^{0}_{R^0},0)|^2 \right] \\  &+ \sum _{k=1}^{n-1} \frac{L^2}{M^{n-k}} \mathbb {E}\! \left[ |U^0_{k}(x+BW^{0}_{R^0})-U^1_{k-1}(x+BW^0_{R^0})|^2 \right] \bigg ]. \end{aligned} \end{aligned}$$

This establishes (117). The proof of Lemma 3.7 is thus completed. $\square$

### Error analysis for MLP approximations

In this subsection we combine the estimates for the biases and variances of MLP approximations from Sect. 3.4 in order to obtain a recursive overall error estimate for MLP approximations, see Lemma 3.8 below. From this result and the a priori estimate in Proposition 2.11 we then obtain a full error analysis for MLP approximations, see Proposition 3.9, Corollary 3.10, and Corollary 3.11 below.

In Lemma 3.8 below we establish a recursive error estimate for MLP approximations. Roughly speaking, we use the bias and variance estimates (see Lemmas 3.6 and 3.7 above) to relate an average approximation error for an MLP approximation at level *n* to average approximation errors for MLP approximations at levels below *n* under certain assumptions on the measures determining the averaging procedures. The choice of these measures will be crucial to obtain appropriate convergence speed of MLP approximations later on.

#### Lemma 3.8

(Recursive overall error estimate) Assume Setting 3.1, let $L \in \mathbb {R}$, $\varepsilon \in (0,\infty )$, assume for all $x \in \mathbb {R}^d$, $v,w \in \mathbb {R}$ that $| f( x, v ) - f( x, w ) | \leqslant L | v - w |$ and $\int _0^{\infty } e^{(\varepsilon -\lambda ) s} \, \mathbb {E}[ |f(x+BW^{0}_s,0)| ]\,ds<\infty$, let $u :\mathbb {R}^d \rightarrow \mathbb {R}$ be measurable, assume for all $x \in \mathbb {R}^d$ that $\int _0^{\infty } e^{-\lambda s} \, \mathbb {E}[ |u(x+BW^{0}_s)| ]\,ds < \infty$ and

121 $$\begin{aligned} \begin{aligned} u(x)&= \frac{1}{\lambda } \mathbb {E}\! \left[ f(x+BW^{0}_{R^0},u(x+BW^{0}_{R^0})) \right] \\  &= \mathbb {E}\! \left[ \int _0^{\infty } e^{-\lambda s} f(x+BW^{0}_s,u(x+BW^{0}_s))\,ds \right] \!, \end{aligned} \end{aligned}$$

and let $\alpha , \beta :\mathcal {B}([0,\infty ) ) \rightarrow [0,\infty )$ be measures which satisfy for all $A \in \mathcal {B}([0,\infty ))$ that $\beta (A) \geqslant \int _A e^{-\lambda s} (\int _{[0,s]} e^{\lambda t}\,\alpha (dt))\,ds$. Then it holds for all $n \in \mathbb {N}$, $\theta \in \Theta$, $x \in \mathbb {R}^d$ that122

#### Proof of Lemma 3.8

Throughout this proof let $\nu _{t,x} :\mathcal {B}(\mathbb {R}^d) \rightarrow [0,\infty )$, $t\in [0,\infty )$, $x \in \mathbb {R}^d$, satisfy for all $t\in [0,\infty )$, $x \in \mathbb {R}^d$, $A \in \mathcal {B}(\mathbb {R}^d)$ that

123 $$\begin{aligned} \nu _{t,x}(A) = \mathbb {P}(x+BW^0_t\in A). \end{aligned}$$

Observe that the triangle inequality ensures for all $n \in \mathbb {N}$, $\theta \in \Theta$, $x \in \mathbb {R}^d$ that124Lemma 3.6, Lemma 3.7, the fact that for all $n\in \mathbb {N}$, $a_1,a_2,\ldots ,a_n\in [0,\infty )$ it holds that $\sqrt{\sum _{k=1}^n a_k} \leqslant \sum _{k=1}^n \sqrt{a_k}$, and the triangle inequality hence prove for all $n \in \mathbb {N}$, $\theta \in \Theta$, $x \in \mathbb {R}^d$ that125This, Items (ii) and (iv) in Lemma 3.2, and [47, Lemma 2.2] (with $(\Omega ,\mathcal {F},\mathbb {P}) \leftarrow (\Omega ,\mathcal {F},\mathbb {P})$, $\mathcal {G} \leftarrow \sigma _{\Omega }( (W^{(1,\vartheta )})_{\vartheta \in \Theta }, (R^{(1,\vartheta )})_{\vartheta \in \Theta } )$, $(S,\mathcal {S}) \leftarrow (\mathbb {R}^d,\mathcal {B}(\mathbb {R}^d))$, $U \leftarrow ( \mathbb {R}^d \times \Omega \ni (y,\omega ) \mapsto |U^1_{k-1}(y,\omega )-u(y)|^2 \in [0,\infty ) )$, $Y \leftarrow ( \Omega \ni \omega \mapsto x + BW^0_{R^0(\omega )}(\omega ) \in \mathbb {R}^d )$ for $x \in \mathbb {R}^d$, $k \in \mathbb {N}$ in the notation of [47, Lemma 2.2]) demonstrate for all $n \in \mathbb {N}$, $\theta \in \Theta$, $x \in \mathbb {R}^d$ that126Hence, we obtain for all $n \in \mathbb {N}$, $\theta \in \Theta$, $x \in \mathbb {R}^d$ that127Item (ii) in Lemma 3.2 and [47, Lemma 2.2] hence ensure for all $n \in \mathbb {N}$, $\theta \in \Theta$, $x \in \mathbb {R}^d$ that128Moreover, note that Items (ii) and (iv) in Lemma 3.2, [47, Lemma 2.2] (with $(\Omega ,\mathcal {F},\mathbb {P}) \leftarrow (\Omega ,\mathcal {F},\mathbb {P})$, $\mathcal {G} \leftarrow \sigma _{\Omega }( (W^{(\theta ,\vartheta )})_{\vartheta \in \Theta }, (R^{(\theta ,\vartheta )})_{\vartheta \in \Theta } )$, $(S,\mathcal {S}) \leftarrow (\mathbb {R}^d,\mathcal {B}(\mathbb {R}^d))$, $U \leftarrow ( \mathbb {R}^d\times \Omega \ni (y,\omega ) \mapsto |U^{\theta }_n(y,\omega ) - u(y) |^2 \in [0,\infty ) )$, $Y \leftarrow (\Omega \ni \omega \mapsto x + BW^{\theta }_{t}(\omega ) \in \mathbb {R}^d)$ for $\theta \in \Theta$, $t \in [0,\infty )$, $x \in \mathbb {R}^d$, $n \in \mathbb {N}$ in the notation of [47, Lemma 2.2]), and (123) prove for all $n \in \mathbb {N}$, $\theta \in \Theta$, $t \in [0,\infty )$, $x \in \mathbb {R}^d$ that129The triangle inequality and (128) hence show for all $n \in \mathbb {N}$, $\theta \in \Theta$, $t \in [0,\infty )$, $x \in \mathbb {R}^d$ that130Combining this with the fact that for all $t,s \in [0,\infty )$, $x \in \mathbb {R}^d$, $A \in \mathcal {B}(\mathbb {R}^d)$ it holds that $\nu _{t+s,x}(A) = \int _{\mathbb {R}^d} \nu _{s,y}(A) \,\nu _{t,x}(dy)$ and Fubini’s theorem proves for all $n \in \mathbb {N}$, $\theta \in \Theta$, $t \in [0,\infty )$, $x \in \mathbb {R}^{d}$ that131The triangle inequality and Fubini’s theorem hence ensure for all $n \in \mathbb {N}$, $\theta \in \Theta$, $x \in \mathbb {R}^d$ that132The assumption that for all $A \in \mathcal {B}([0,\infty ))$ it holds that

133 $$\begin{aligned} \textstyle \beta (A) \geqslant \int _A e^{-\lambda s} (\int _{[0,s]} e^{\lambda t}\,\alpha (dt))\, ds \end{aligned}$$

hence ensures for all $n \in \mathbb {N}$, $\theta \in \Theta$, $x \in \mathbb {R}^d$ that134This establishes (122). The proof of Lemma 3.8 is thus completed. $\square$

In the next result we make appropriate choices for the measures $\alpha$ and $\beta$ appearing in Lemma 3.8 above. This allows us to *close* the estimates in Lemma 3.8, get into a setting in which a Gronwall type argument can be applied, and finally establish an overall error estimate for MLP approximations, see Proposition 3.9 below. An error estimate for MLP approximations for related semilinear parabolic PDEs can be found in [47, Theorem 3.5].

#### Proposition 3.9

(Overall error estimate) Assume Setting 3.1, let $L \in (0,\lambda )$, assume for all $x \in \mathbb {R}^d$, $v,w \in \mathbb {R}$ that $| f( x, v ) - f( x, w ) | \leqslant L | v - w |$ and $\int _0^{\infty } e^{(L-\lambda ) s} \, \mathbb {E}[ |f(x+BW^{0}_s,0)| ]\,ds<\infty$, let $u :\mathbb {R}^d \rightarrow \mathbb {R}$ be measurable, and assume for all $x \in \mathbb {R}^d$ that $\int _0^{\infty } e^{(L-\lambda ) s} \, \mathbb {E}[ |u(x+BW^{0}_s)|^2 ]\,ds < \infty$ and

135 $$\begin{aligned} \begin{aligned}&u(x) = \frac{1}{\lambda } \mathbb {E}\! \left[ f(x+BW^{0}_{R^0},u(x+BW^{0}_{R^0})) \right] \\  &= \mathbb {E}\! \left[ \int _0^{\infty } e^{-\lambda s} f(x+BW^{0}_s,u(x+BW^{0}_s))\,ds \right] \!. \end{aligned} \end{aligned}$$

Then it holds for all $n \in \mathbb {N}_0$, $\theta \in \Theta$, $x \in \mathbb {R}^d$ that136

#### Proof of Proposition 3.9

Throughout this proof let $C = 1 + (1+\sqrt{M})\sqrt{\frac{L}{\lambda }}$ and let $\delta _0 :\mathcal {B}([0,\infty )) \rightarrow [0,\infty )$ satisfy for all $A \in \mathcal {B}([0,\infty ))$ that

137 $$\begin{aligned} \delta _0(A) = {\left\{ \begin{array}{ll} 1 &  :0 \in A \\ 0 &  :0 \notin A. \end{array}\right. } \end{aligned}$$

Observe that

(i) it holds for all $A \in \mathcal {B}([0,\infty ))$ that
  138 $$\begin{aligned} \int _A e^{-\lambda s} \int _{[0,s]} e^{\lambda t} \,\delta _0(dt) \,ds = \int _A e^{-\lambda s} \,ds \leqslant \int _A e^{(L-\lambda ) s}\,ds \end{aligned}$$
  and
(ii) it holds for all $A \in \mathcal {B}([0,\infty ))$ that
  139 $$\begin{aligned} \int _A e^{-\lambda s} \int _{[0,s]} e^{\lambda t}\, e^{(L-\lambda )t} \,dt \,ds = \int _A e^{-\lambda s} \frac{e^{Ls}-1}{L}\,ds \leqslant \frac{1}{L} \int _A {e^{(L-\lambda ) s}}\,ds. \end{aligned}$$

Note that Item (ii) and Lemma 3.8 (with $\varepsilon \leftarrow L$, $L \leftarrow L$, $u \leftarrow u$, $\alpha \leftarrow ( \mathcal {B}([0,\infty )) \ni A \mapsto \int _A e^{(L-\lambda )t}\,dt \in [0,\infty ) )$, $\beta \leftarrow ( \mathcal {B}([0,\infty )) \ni A \mapsto \frac{1}{L} \int _A e^{(L-\lambda )t}\,dt \in [0,\infty ))$ in the notation of Lemma 3.8) ensure for all $n \in \mathbb {N}$, $x \in \mathbb {R}^d$ that140For the next step let $\eta ^{(x)}_n \in [0,\infty ]$, $n \in \mathbb {N}_0$, $x \in \mathbb {R}^d$, satisfy for all $n \in \mathbb {N}_0$, $x \in \mathbb {R}^d$ that141and let $a_1^{(x)} \in [0,\infty )$, $x \in \mathbb {R}^d$, and $a_2^{(x)} \in [0,\infty ]$, $x \in \mathbb {R}^d$, satisfy for all $x \in \mathbb {R}^d$ that142and $a_2^{(x)} = ( 1 + \sqrt{M} ) \frac{ \sqrt{L} }{ \sqrt{\lambda } }$. Note that (140) ensures for all $n \in \mathbb {N}$, $x \in \mathbb {R}^d$ that $\eta ^{(x)}_n \leqslant a_1^{(x)} + a_2^{(x)} \sum _{k=0}^{n-1} \eta ^{(x)}_k$. The discrete Gronwall inequality therefore proves for all $n \in \mathbb {N}$, $x \in \mathbb {R}^d$ that

143 $$\begin{aligned} \eta ^{(x)}_n \leqslant ( a^{(x)}_1 + a^{(x)}_2\eta ^{(x)}_0 ) ( 1 + a_2^{(x)})^{n-1}. \end{aligned}$$

Next observe that Item (ii) in Proposition 2.11 demonstrates for all $x \in \mathbb {R}^d$ that144This and (143) ensure for all $n \in \mathbb {N}_0$, $x \in \mathbb {R}^d$ that . Hence, we obtain for all $n \in \mathbb {N}_0$, $x \in \mathbb {R}^d$ that145Next note that Item (i) and Lemma 3.8 (with $\varepsilon \leftarrow L$, $L \leftarrow L$, $u \leftarrow u$, $\alpha \leftarrow \delta _0$, $\beta \leftarrow (\mathcal {B}([0,\infty )) \ni A \mapsto \int _A e^{(L-\lambda )s}\,ds \in [0,\infty ))$ in the notation of Lemma 3.8) imply for all $n \in \mathbb {N}$, $\theta \in \Theta$, $x \in \mathbb {R}^d$ that146This and (145) demonstrate for all $n \in \mathbb {N}$, $\theta \in \Theta$, $x \in \mathbb {R}^d$ thatHence, we obtain for all $n \in \mathbb {N}$, $\theta \in \Theta$, $x \in \mathbb {R}^d$ that147The fact that for all $q \in \mathbb {R}$, $n \in \mathbb {N}$ it holds that $(q-1) \sum _{k=0}^{n-1} q^k = q^n-1$ therefore implies for all $n \in \mathbb {N}$, $\theta \in \Theta$, $x \in \mathbb {R}^d$ that148The fact that $\frac{\sqrt{L\lambda }}{\sqrt{L\lambda }-L}>1$ hence implies for all $n \in \mathbb {N}$, $\theta \in \Theta$, $x \in \mathbb {R}^d$ that149Combining this with the fact that for all $\theta \in \Theta$, $x \in \mathbb {R}^d$ it holds that $U^{\theta }_0(x) = 0$ and Item (iii) in Proposition 2.11 establishes (136). The proof of Proposition 3.9 is thus completed. $\square$

Our proof of Proposition 3.9 required that the Lipschitz constant *L* is positive. In Corollary 3.10 below we employ a limiting argument to remove this hypothesis. Combining Proposition 3.9 with Corollary 3.10 will lead to the full error estimate for MLP approximations in Corollary 3.11 below.

#### Corollary 3.10

Assume Setting 3.1, let $\varepsilon \in (0,\infty )$, assume for all $x \in \mathbb {R}^d$, $v \in \mathbb {R}$ that $f(x,v) = f(x,0)$ and $\int _0^{\infty } e^{(\varepsilon -\lambda )s}\, \mathbb {E}[ |f(x+BW^{0}_s,0)|^2 ]\,ds < \infty$, let $u :\mathbb {R}^d \rightarrow \mathbb {R}$ be measurable, and assume for all $x \in \mathbb {R}^d$ that

150 $$\begin{aligned} \begin{aligned} u(x)&= \mathbb {E}\! \left[ \int _0^{\infty } e^{-\lambda s} f(x+BW^{0}_s,u(x+BW^{0}_s))\,ds \right] \\  &= \mathbb {E}\! \left[ \int _0^{\infty } e^{-\lambda s} f(x+BW^{0}_s,0)\,ds \right] \!. \end{aligned} \end{aligned}$$

Then it holds for all $n \in \mathbb {N}_0$, $\theta \in \Theta$, $x \in \mathbb {R}^d$ that151

#### Proof of Corollary 3.10

Throughout this proof let $\nu _{t,x} :\mathcal {B}(\mathbb {R}^d) \rightarrow [0,\infty )$, $t\in [0,\infty )$, $x \in \mathbb {R}^d$, satisfy for all $t \in [0,\infty )$, $x \in \mathbb {R}^d$, $A \in \mathcal {B}(\mathbb {R}^d)$ that

152 $$\begin{aligned} \nu _{t,x}(A) = \mathbb {P}(x+BW^0_t\in A). \end{aligned}$$

Observe that the Cauchy–Schwarz inequality and (150) ensure for all $y \in \mathbb {R}^d$ that $|u(y)|^2 \leqslant \frac{1}{\lambda } \int _0^{\infty } e^{-\lambda s} \, \mathbb {E}\! \left[ |f(y+BW^0_s,0)|^2 \right] \!\,ds$. Fubini’s theorem therefore shows for all $x \in \mathbb {R}^d$ that

153 $$\begin{aligned} \begin{aligned}&\int _0^{\infty } e^{(\varepsilon -\lambda )t} \, \mathbb {E}\! \left[ |u(x+BW^0_t)|^2 \right] \!\,dt = \int _0^{\infty } e^{(\varepsilon -\lambda )t} \int _{\mathbb {R}^d} |u(y)|^2\,\nu _{t,x}(dy)\,dt \\&\leqslant \int _0^{\infty } e^{(\varepsilon -\lambda )t}\, \int _{\mathbb {R}^d} \frac{1}{\lambda } \int _0^{\infty } e^{-\lambda s}\, \mathbb {E}\! \left[ |f(y+BW^0_s,0)|^2 \right] \!\,ds\,\nu _{t,x}(dy)\,dt \\&= \frac{1}{\lambda } \int _0^{\infty } e^{(\varepsilon -\lambda )t} \int _0^{\infty } e^{-\lambda s} \, \int _{\mathbb {R}^d} \mathbb {E}\! \left[ |f(y+BW^0_s,0)|^2 \right] \!\,\nu _{t,x}(dy)\,ds\,dt. \end{aligned} \end{aligned}$$

The fact that for all $t,s \in [0,\infty )$, $x \in \mathbb {R}^d$, $A \in \mathcal {B}(\mathbb {R}^d)$ it holds that $\nu _{t+s,x}(A) = \int _{\mathbb {R}^d} \nu _{s,y}(A)\,\nu _{t,x}(dy)$ and Fubini’s theorem hence ensure that for all $x \in \mathbb {R}^d$ it holds that

154 $$\begin{aligned} \begin{aligned}&\int _0^{\infty } e^{(\varepsilon -\lambda )t} \, \mathbb {E}\! \left[ |u(x+BW^0_t)|^2 \right] \!\,dt \\  &\leqslant \frac{1}{\lambda } \int _0^{\infty } e^{(\varepsilon -\lambda )t} \int _0^{\infty } e^{-\lambda s} \, \mathbb {E}\! \left[ |f(x+BW^0_{t+s},0)|^2 \right] \!\,ds\,dt \\&= \frac{1}{\lambda } \int _0^{\infty } e^{\varepsilon t} \int _t^{\infty } e^{-\lambda s} \, \mathbb {E}\! \left[ |f(x+BW^0_{s},0)|^2 \right] \!\,ds\,dt \\&= \frac{1}{\lambda } \int _0^{\infty } \left[ \int _0^s e^{\varepsilon t} \,dt\right] \!\, e^{-\lambda s} \, \mathbb {E}\! \left[ |f(x+BW^0_s,0)|^2 \right] \!\,ds \\&\leqslant \frac{1}{\varepsilon \lambda } \int _0^{\infty } e^{(\varepsilon -\lambda )s} \, \mathbb {E}\! \left[ |f(x+BW^0_s,0)|^2 \right] \!\,ds < \infty . \end{aligned} \end{aligned}$$

Proposition 3.9 therefore proves for all $L \in (0,\min \{\varepsilon ,\lambda \})$, $n \in \mathbb {N}_0$, $\theta \in \Theta$, $x \in \mathbb {R}^d$ that155Lebesgue’s dominated convergence theorem hence ensures for all $n \in \mathbb {N}_0$, $\theta \in \Theta$, $x \in \mathbb {R}^d$ that156This establishes (151). The proof of Corollary 3.10 is thus completed. $\square$

Combining Proposition 3.9 with Corollary 3.10 establishes the full error estimate for MLP approximations, see Corollary 3.11 below.

#### Corollary 3.11

Assume Setting 3.1, let $L \in [0,\lambda )$, $\eta \in (0,\infty )$, assume for all $x \in \mathbb {R}^d$, $v,w \in \mathbb {R}$ that $| f( x, v ) - f( x, w ) | \leqslant L | v - w |$ and $\int _0^{\infty } e^{(\max \{L,\eta \}-\lambda ) s} \, \mathbb {E}[ | f( x + BW^0_s, 0) |^2 ] \, ds < \infty$, let $u :\mathbb {R}^d \rightarrow \mathbb {R}$ be measurable, and assume for all $x \in \mathbb {R}^d$ that $\int _0^{\infty } e^{(L-\lambda )s} \, \mathbb {E}[ |u(x+BW^0_s)|^2 ]\, ds < \infty$ and

157 $$\begin{aligned} \begin{aligned} u(x)&= \frac{1}{\lambda } \mathbb {E}\! \left[ f(x+BW^0_{R^0},u(x+BW^0_{R^0})) \right] \\  &= \int _0^{\infty } e^{-\lambda s}\, \mathbb {E}\! \left[ f(x+BW^0_s,u(x+BW^0_s)) \right] \!\,ds. \end{aligned} \end{aligned}$$

Then it holds for all $\theta \in \Theta$, $n \in \mathbb {N}_0$, $x \in \mathbb {R}^d$ that158

#### Proof of Corollary 3.11

Corollary 3.11 is an immediate consequence of Proposition 3.9 and Corollary 3.10. A detailed proof can be found, e.g., in the extended preprint version [7, Corollary 3.11] of this article. $\square$

In Corollary 3.12 below we provide a version of Corollary 3.11 in spaces of functions with growth controlled by a Lyapunov type function.

#### Corollary 3.12

Assume Setting 3.1, let $L,\rho \in [0,\infty )$, assume that $\lambda \in (L+2\rho ,\infty )$, let $\left\| \cdot \right\| :\mathbb {R}^d \rightarrow [0,\infty )$ be the standard norm on $\mathbb {R}^d$, let $u \in C(\mathbb {R}^d,\mathbb {R})$, $V \in C^2(\mathbb {R}^d,(0,\infty ))$ satisfy for all $x \in \mathbb {R}^d$, $v,w \in \mathbb {R}$ that $| f(x,v) - f(x,w) | \leqslant L | v-w |$ and

159 $$\begin{aligned} {\text {Trace}}(BB^{*}({\text {Hess}} V)(x))+\tfrac{ \left\| B^{*}(\nabla V)(x) \right\| ^2}{V(x)} \leqslant 2\rho V(x), \end{aligned}$$

assume that $\sup _{r\in (0,\infty )} [\inf _{ \left\| x \right\| >r} V(x)] = \infty$ and $\inf _{r\in (0,\infty )} [ \sup _{ \left\| x \right\| >r} (\frac{|f(x,0)|+|u(x)|}{V(x)}) ] = 0$, and assume that *u* is a viscosity solution of

160 $$\begin{aligned} \lambda u(x) - \tfrac{1}{2}{\text {Trace}}\!\big (BB^{*}({\text {Hess}} u)(x)\big ) = f(x,u(x)) \end{aligned}$$

for $x \in \mathbb {R}^d$ (cf. Proposition 2.7). Then it holds for all $\theta \in \Theta$, $n \in \mathbb {N}_0$, $x \in \mathbb {R}^d$ that161

#### Proof of Corollary 3.12

Throughout this proof let $c\in \mathbb {R}$ satisfy for all $x \in \mathbb {R}^d$ that $|u(x)| \leqslant c V(x)$. Note that (159) ensures that

(I) it holds for all $x \in \mathbb {R}^d$ that $\frac{1}{2}{\text {Trace}}(BB^{*}({\text {Hess}} V)(x)) \leqslant \rho V(x)$ and
(II) it holds for all $x \in \mathbb {R}^d$ that
  162 $$\begin{aligned} \begin{aligned}&\tfrac{1}{2}{\text {Trace}}\!\big (BB^{*}({\text {Hess}} V^2)(x) \big ) \\&= \tfrac{1}{2}{\text {Trace}}\!\big (BB^{*}( 2 V(x) ({\text {Hess}} V)(x) + 2 (\nabla V)(x)((\nabla V)(x))^\top ) \big ) \\  &= V(x) \left[ {\text {Trace}}\!\big (BB^{*}({\text {Hess}} V)(x) \big ) + \tfrac{ \left\| B^{*}(\nabla V)(x) \right\| ^2}{V(x)} \right] \leqslant 2 \rho [V(x)]^2. \end{aligned} \end{aligned}$$

Note that item (I) and Proposition 2.7 demonstrate for all $x \in \mathbb {R}^d$ that

163 $$\begin{aligned} u(x) = \mathbb {E}\! \left[ \int _0^{\infty } e^{-\lambda s} f(x+BW^{0}_s,u(x+BW^{0}_s))\,ds \right] \!. \end{aligned}$$

Next note that [6, Lemmas 3.1 and 3.2] and Item (II) guarantee for all $t \in [0,\infty )$, $x \in \mathbb {R}^d$ that

164 $$\begin{aligned} \mathbb {E}\big [ |V(x+BW^0_t)|^2 \big ] \leqslant e^{2\rho t} [V(x)]^2. \end{aligned}$$

The fact that $\sup _{y\in \mathbb {R}^d} (\frac{|f(y,0)|}{V(y)}) < \infty$ hence implies for all $x \in \mathbb {R}^d$, $\eta \in [0,\infty )$ with $L+2\rho +\eta < \lambda$ that

165 $$\begin{aligned} \begin{aligned}&\int _0^{\infty } e^{(L+\eta -\lambda )s} \, \mathbb {E}\! \left[ |f(x+BW^0_s,0)|^2 \right] \!\,ds \\&\leqslant \int _0^{\infty } e^{(L+\eta -\lambda )s} \left[ \sup _{y\in \mathbb {R}^d}\left( \frac{|f(y,0)|}{V(y)} \right) \right] ^2 \, \mathbb {E}\! \left[ |V(x+BW^0_s)|^2 \right] \!\,ds \\  &\leqslant \frac{|V(x)|^2}{\lambda -(L+\eta +2\rho )} \left[ \sup _{y\in \mathbb {R}^d}\left( \frac{|f(y,0)|}{V(y)} \right) \right] ^2 < \infty . \end{aligned} \end{aligned}$$

In addition, note that the fact that for all $x \in \mathbb {R}^d$ it holds that $|u(x)| \leqslant c V(x)$ and (164) prove for all $x \in \mathbb {R}^d$ that

166 $$\begin{aligned} \begin{aligned}&\int _{0}^{\infty } e^{(L-\lambda )s}\, \mathbb {E}\! \left[ |u(x+BW^0_s)|^2 \right] \!\,ds \\  &\leqslant \int _0^{\infty } e^{(L-\lambda )s}\,c^2 \, \mathbb {E}\! \left[ |V(x+BW^0_s)|^2 \right] \!\,ds \\&\leqslant c^2 |V(x)|^2\int _0^{\infty } e^{(L+2\rho -\lambda )s} \,ds = \frac{[cV(x)]^2}{\lambda -(L+2\rho )} < \infty . \end{aligned} \end{aligned}$$

This, (163), (165), and Corollary 3.11 assure for all $\theta \in \Theta$, $n \in \mathbb {N}_0$, $x \in \mathbb {R}^d$ that167This establishes (161). The proof of Corollary 3.12 is thus completed. $\square$

In Corollary 3.13 below we concretize Corollary 3.12 by choosing Lyapunov type functions related to polynomial growth. In addition, we consider semilinear elliptic PDE given in a slightly different form, see also Corollary 2.10 above.

#### Corollary 3.13

Let $d,M \in \mathbb {N}$, $B \in \mathbb {R}^{d\times d}$, $L \in \mathbb {R}$, $\lambda \in (L,\infty )$, $\Theta = \cup _{n\in \mathbb {N}} \mathbb {Z}^n$, $f \in C(\mathbb {R}^d\times \mathbb {R},\mathbb {R})$ satisfy for all $x \in \mathbb {R}^d$, $v,w \in \mathbb {R}$ that

168 $$\begin{aligned} | f(x,v) - f(x,w) - \lambda ( v - w ) | \leqslant L | v-w |, \end{aligned}$$

assume that *f* is at most polynomially growing, let $\left\| \cdot \right\| :\mathbb {R}^d \rightarrow [0,\infty )$ be the standard norm on $\mathbb {R}^d$, let $( \Omega , \mathcal {F}, \mathbb {P})$ be a probability space, let $W^{\theta } :[0,\infty ) \times \Omega \rightarrow \mathbb {R}^d$, $\theta \in \Theta$, be i.i.d. standard Brownian motions, let $R^{\theta } :\Omega \rightarrow [0,\infty )$, $\theta \in \Theta$, be i.i.d. random variables, assume for all $t\in [0,\infty )$ that $\mathbb {P}(R^0 \geqslant t) = e^{-\lambda t}$, assume that $(R^{\theta })_{\theta \in \Theta }$ and $(W^{\theta })_{\theta \in \Theta }$ are independent, let $U^{\theta }_{n} = (U^{\theta }_{n}(x))_{x\in \mathbb {R}^d}:\mathbb {R}^d\times \Omega \rightarrow \mathbb {R}$, $\theta \in \Theta$, $n\in \mathbb {N}_0$, satisfy for all $\theta \in \Theta$, $n \in \mathbb {N}$, $x \in \mathbb {R}^d$ that $U^{\theta }_{0}(x) = 0$ and

169 $$\begin{aligned} \begin{aligned}&U^{\theta }_{n}(x) = \frac{-1}{\lambda M^n} \left[ \sum _{m=1}^{M^n} f(x+BW^{(\theta ,0,m)}_{R^{(\theta ,0,m)}},0) \right] + \sum _{k=1}^{n-1} \frac{1}{\lambda M^{n-k}} \\  &\cdot \Bigg [ \sum _{m=1}^ {M^{n-k}} \bigg ( \lambda \Big [ U^{(\theta ,k,m)}_{k}(x+BW^{(\theta ,k,m)}_{R^{(\theta ,k,m)}}) - U^{(\theta ,k,-m)}_{k-1}(x+BW^{(\theta ,k,m)}_{R^{(\theta ,k,m)}}) \Big ] \\  &- \Big [ f\!\left( x+BW^{(\theta ,k,m)}_{R^{(\theta ,k,m)}}, U^{(\theta ,k,m)}_{k}(x+BW^{(\theta ,k,m)}_{R^{(\theta ,k,m)}}) \right) \\&- f\!\left( x+BW^{(\theta ,k,m)}_{R^{(\theta ,k,m)}}, U^{(\theta ,k,-m)}_{k-1}(x+BW^{(\theta ,k,m)}_{R^{(\theta ,k,m)}}) \right) \Big ] \bigg ) \Bigg ], \end{aligned} \end{aligned}$$

and let $u \in \{ v \in C(\mathbb {R}^d,\mathbb {R}) :( \forall \,\varepsilon \in (0,\infty ) :[ \sup _{x=(x_1,\ldots ,x_d)\in \mathbb {R}^d} ( | v(x) | \exp ( - \varepsilon \sum _{i=1}^d |x_i| ) ) ] < \infty ) \}$ be a viscosity solution of

170 $$\begin{aligned} \tfrac{1}{2}{\text {Trace}}\!\big (BB^{*}({\text {Hess}} u)(x)\big ) = f(x,u(x)) \end{aligned}$$

for $x \in \mathbb {R}^d$ (cf. Corollary 2.10). Then it holds for all $n \in \mathbb {N}_0$, $\theta \in \Theta$, $x \in \mathbb {R}^d$, $\varepsilon \in (0,\infty )$ with $2 (\varepsilon ^2+\varepsilon d) [\sup _{y\in \mathbb {R}^d{{\setminus }}\{0\}} ( \left\| By \right\| \left\| y \right\| ^{-1})]^2 < \lambda - L$ that171

#### Proof of Corollary 3.13

Throughout this proof let $g :\mathbb {R}^d \times \mathbb {R}\rightarrow \mathbb {R}$ satisfy for all $x \in \mathbb {R}^d$, $v \in \mathbb {R}$ that $g(x,v) = \lambda v - f(x,v)$, let $\beta \in [0,\infty )$ satisfy $\beta = [\sup _{y\in \mathbb {R}^d{{\setminus }}\{0\}} (\frac{ \left\| By \right\| }{ \left\| y \right\| })]^2$, and let $V_{\varepsilon } :\mathbb {R}^d \rightarrow (0,\infty )$, $\varepsilon \in (0,\infty )$, satisfy for all $\varepsilon \in (0,\infty )$, $x \in \mathbb {R}^d$ that . Observe that Lemma 2.9 implies for all $\varepsilon \in (0,\infty )$, $x \in \mathbb {R}^d$ that

172 $$\begin{aligned} {\text {Trace}}\!\big ( BB^{*}({\text {Hess}} V_{\varepsilon })(x) \big ) + \frac{ \left\| B^{*}(\nabla V_{\varepsilon })(x) \right\| ^2}{V_{\varepsilon }(x)} \leqslant 2(\varepsilon ^2 + \varepsilon d) \beta V_{\varepsilon }(x). \end{aligned}$$

In addition, note that the fact that for all $x \in \mathbb {R}^d$, $v \in \mathbb {R}$ it holds that $g(x,v) = \lambda v - f(x,v)$ and (169) ensure for all $\theta \in \Theta$, $n \in \mathbb {N}$, $x \in \mathbb {R}^d$ that

173 $$\begin{aligned} \begin{aligned}&U^{\theta }_{n}(x) \\  &= \sum _{k=1}^{n-1} \frac{1}{\lambda M^{n-k}} \Bigg [ \sum _{m=1}^{M^{n-k}} \bigg ( g\!\left( x+BW^{(\theta ,k,m)}_{R^{(\theta ,k,m)}}, U^{(\theta ,k,m)}_{k}(x+BW^{(\theta ,k,m)}_{R^{(\theta ,k,m)}}) \right) \\&- g\!\left( x+BW^{(\theta ,k,m)}_{R^{(\theta ,k,m)}}, U^{(\theta ,k,-m)}_{k-1}(x+BW^{(\theta ,k,m)}_{R^{(\theta ,k,m)}}) \right) \bigg ) \Bigg ] \\  &+ \frac{1}{\lambda M^n} \left[ \sum _{m=1}^{M^n} g(x+BW^{(\theta ,0,m)}_{R^{(\theta ,0,m)}},0) \right] \!. \end{aligned} \end{aligned}$$

Moreover, observe that the fact that for all $x \in \mathbb {R}^d$, $v \in \mathbb {R}$ it holds that $g(x,v) = \lambda v - f(x,v)$ and (170) ensure that *u* is a viscosity solution of

174 $$\begin{aligned} \lambda u(x) - \tfrac{1}{2} {\text {Trace}}\!\big ( BB^{*}({\text {Hess}} u)(x) \big ) = g(x,u(x)) \end{aligned}$$

for $x \in \mathbb {R}^d$. In addition, note that the fact that for all $\varepsilon \in (0,\infty )$ it holds that $\sup _{ x \in \mathbb {R}^d } ( \frac{ |g(x,0)|+|u(x)| }{ V_{\varepsilon }(x) } ) < \infty$ guarantees for all $\varepsilon \in (0,\infty )$ that

175 $$\begin{aligned} \textstyle \inf _{ r \in (0,\infty ) } [ \sup _{ \left\| x \right\| >r } ( \frac{ |g(x,0)|+|u(x)| }{ V_{\varepsilon }(x) } ) ] = 0. \end{aligned}$$

Corollary 3.12, (172), (173), and (174) therefore demonstrate for all $\theta \in \Theta$, $n \in \mathbb {N}_0$, $x \in \mathbb {R}^d$, $\varepsilon \in (0,\infty )$ with $2(\varepsilon ^2 + \varepsilon d ) \beta < \lambda - L$ that176This and the fact that for all $y \in \mathbb {R}^d$ it holds that $-f(y,0) = g(y,0)$ establish (171). The proof of Corollary 3.13 is thus complete. $\square$

### Computational cost analysis for MLP approximations

In this subsection we provide an upper bound for the computational cost to sample realizations of MLP approximations.

We start by proving a Gronwall type inequality, see Lemma 3.14 below. Lemma 3.14 will allow us to estimate the computational cost to sample realizations of MLP approximations. Our proof employs similar arguments as the proof of [47, Lemma 3.6].

#### Lemma 3.14

Let $\alpha ,\beta \in [0,\infty )$, $M \in [1,\infty )$, $(C_n)_{n\in \mathbb {N}_0} \subseteq [0,\infty )$ satisfy for all $n \in \mathbb {N}$ that

177 $$\begin{aligned} C_n \leqslant \alpha M^n + \sum _{k=1}^{n-1} M^{n-k} ( \beta + C_k + C_{k-1} ). \end{aligned}$$

Then it holds for all $n \in \mathbb {N}$ that

178 $$\begin{aligned} C_n \leqslant \left[ \frac{\alpha + \beta + C_0 }{2}\right] (2M+1)^n \leqslant \left[ \frac{\alpha + \beta + C_0 }{2}\right] (3M)^n. \end{aligned}$$

#### Proof of Lemma 3.14

Throughout this proof let $c_n \in [0,\infty )$, $n \in \mathbb {N}_0$, satisfy for all $n \in \mathbb {N}_0$ that $c_n = \frac{C_n}{M^n}$ and let $S_n \in [0,\infty )$, $n \in \mathbb {N}_0$, satisfy for all $n \in \mathbb {N}_0$ that179Note that (177), the fact that $(c_n)_{n\in \mathbb {N}_0} \subseteq [0,\infty )$, and the assumption that $M \geqslant 1$ ensure for all $n \in \mathbb {N}$ that180Combining this with (179) proves for all $n \in \mathbb {N}\cap [2,\infty )$ that

.

This implies for all $n \in \mathbb {N}$ that $S_{n} \leqslant (2+\frac{1}{M})^{n-1} S_1$. Combining this with (180) and the assumption that $\beta \geqslant 0$ demonstrates for all $n \in \mathbb {N}\cap [2,\infty )$ that181Next observe that (180) implies that $c_1 \leqslant \alpha$. Combining this with (179) ensures that182This and (181) show for all $n \in \mathbb {N}\cap [2,\infty )$ that183Combining this with the fact that  and the fact that for all $n \in \mathbb {N}_0$ it holds that $C_n = c_n M^n$ establishes (178). The proof of Lemma 3.14 is thus complete. $\square$

In Lemma 3.15 below we establish an elementary and auxiliary result which will allow us in the next section to estimate the overall complexity of MLP approximations by comparing the convergence speed of MLP approximations with the computational cost to sample realizations of MLP approximations.

#### Lemma 3.15

Let $m \in \mathbb {N}_0$, $\alpha ,\beta ,\kappa _1,\kappa _2 \in \mathbb {R}$ satisfy $0< \alpha< 1 < \beta$, let $c_n \in [0,\infty )$, $n \in \mathbb {N}_0 \cap [m,\infty )$, let $e_n \in [0,\infty )$, $n \in \mathbb {N}_0 \cap [m,\infty )$, assume for all $n \in \mathbb {N}_0 \cap [m,\infty )$ that $e_n \leqslant \kappa _1 \alpha ^n$ and $c_n \leqslant \kappa _2 \beta ^n$, and let $N :(0,1] \rightarrow \mathbb {N}_0 \cap [m,\infty )$ satisfy for all $\varepsilon \in (0,1]$ that

184 $$\begin{aligned} N_{\varepsilon } = \min \{n\in \mathbb {N}_0\cap [m,\infty ) :\kappa _1 \alpha ^n \leqslant \varepsilon \}. \end{aligned}$$

Then it holds for all $\varepsilon \in (0,1]$ that $e_{N_{\varepsilon }} \leqslant \varepsilon$ and

185 $$\begin{aligned} c_{N_{\varepsilon }} \leqslant \kappa _2 \max \left\{ \beta ^m, \beta \kappa _1^{(\frac{\ln (\beta )}{\ln (1/\alpha )})} \right\} \left[ \frac{1}{\varepsilon } \right] ^{(\frac{\ln (\beta )}{\ln (1/\alpha )})}. \end{aligned}$$

#### Proof of Lemma 3.15

First, observe that (184) ensures for all $\varepsilon \in (0,1]$ that $e_{N_{\varepsilon }} \leqslant \varepsilon$. Moreover, note that (184) guarantees for all $\varepsilon \in (0,1]$ with $N_{\varepsilon } \geqslant m + 1$ that $\kappa _1 \alpha ^{N_{\varepsilon }-1} > \varepsilon$. Hence, we obtain for all $\varepsilon \in (0,1]$ with $N_{\varepsilon } \geqslant m + 1$ that

.

This implies for all $\varepsilon \in (0,1]$ with $N_{\varepsilon } \geqslant m + 1$ that186Next note that the assumption that for all $n \in \mathbb {N}_0 \cap [m,\infty )$ it holds that $c_n \leqslant \kappa _2 \beta ^n$ and the fact that  ensure that for all $\varepsilon \in (0,1]$ with $N_{\varepsilon } = m$ it holds that

187 $$\begin{aligned} c_{N_{\varepsilon }} = c_m \leqslant \kappa _2 \beta ^m \leqslant \kappa _2 \beta ^m \left[ \frac{1}{\varepsilon } \right] ^{(\frac{\ln (\beta )}{\ln (1/\alpha )})}. \end{aligned}$$

This and (186) establish (185). The proof of Lemma 3.15 is thus complete. $\square$

### Overall complexity analysis for MLP approximations

In this subsection we combine the error analysis for MLP approximations established in Sect. 3.5 with the computational cost analysis for MLP approximations established in Sect. 3.6 to obtain an overall complexity analysis for the proposed MLP approximation schemes, see Theorem 3.16 and Corollary 3.17 below.

#### Theorem 3.16

Let $\kappa ,L,\mathfrak {n},p,q,r,s \in [0,\infty )$, $\lambda \in (L,\infty )$, $M \in \mathbb {N}\cap ( (\sqrt{\lambda }+\sqrt{L})^2 (\sqrt{\lambda }-\sqrt{L})^{-2}, \infty )$, , $\Theta = \cup _{n\in \mathbb {N}} \mathbb {Z}^n$, let $u_d \in C(\mathbb {R}^d,\mathbb {R})$, $d \in \mathbb {N}$, let $B_d \in \mathbb {R}^{d\times d}$, $d \in \mathbb {N}$, let $f_d \in C(\mathbb {R}^d\times \mathbb {R},\mathbb {R})$, $d \in \mathbb {N}$, assume for every $d \in \mathbb {N}$ that $u_d$ is a viscosity solution of

188 $$\begin{aligned} \tfrac{1}{2}{\text {Trace}}(B_d(B_d)^{*}({\text {Hess}} u_d)(x)) = f_d(x,u_d(x)) \end{aligned}$$

for $x \in \mathbb {R}^d$, let $( \Omega , \mathcal {F}, \mathbb {P})$ be a probability space, let $W^{d,\theta } :[0,\infty ) \times \Omega \rightarrow \mathbb {R}^d$, $\theta \in \Theta$, $d \in \mathbb {N}$, be i.i.d. standard Brownian motions, let $R^{\theta } :\Omega \rightarrow [0,\infty )$, $\theta \in \Theta$, be i.i.d. random variables, assume that $(R^{\theta })_{\theta \in \Theta }$ and $(W^{d,\theta })_{(d,\theta )\in \mathbb {N}\times \Theta }$ are independent, let $\left\| \cdot \right\| :(\cup _{d\in \mathbb {N}} \mathbb {R}^d) \rightarrow [0,\infty )$ satisfy for all $d \in \mathbb {N}$, $x = (x_1,\ldots ,x_d) \in \mathbb {R}^d$ that , assume for all $d \in \mathbb {N}$, $\varepsilon \in (0,\infty )$, $x \in \mathbb {R}^d$, $v,w \in \mathbb {R}$ that $\Vert B_d x \Vert \leqslant \kappa d^r \Vert x \Vert$, $| f_d(x,0) | \leqslant \kappa d^{s}( 1 + \left\| x \right\| ^p )$, $| f_d(x,v) - f_d(x,w) - \lambda ( v - w ) | \leqslant L | v-w |$, $\sup _{y\in \mathbb {R}^d} [|u_d(y)|\exp (-\varepsilon \Vert y \Vert )] < \infty$, and $\mathbb {P}(R^0 \geqslant \varepsilon ) = e^{-\lambda \varepsilon }$, let $U^{d,\theta }_{n} = (U^{d,\theta }_{n}(x))_{x\in \mathbb {R}^d}:\mathbb {R}^d\times \Omega \rightarrow \mathbb {R}$, $\theta \in \Theta$, $d, n \in \mathbb {N}_0$, satisfy for all $d,n \in \mathbb {N}$, $\theta \in \Theta$, $x \in \mathbb {R}^d$ that $U^{d,\theta }_{0}(x) = 0$ and

189 $$\begin{aligned} U^{d,\theta }_{n}(x)&= \frac{-1}{\lambda M^n} \left[ \sum _{m=1}^{M^n} f_d(x+B_dW^{d,(\theta ,0,m)}_{R^{(\theta ,0,m)}},0) \right] \nonumber \\&+ \sum _{k=1}^{n-1} \frac{1}{\lambda M^{n-k}} \Bigg [ \sum _{m=1}^ {M^{n-k}} \bigg ( \lambda \Big [ U^{d,(\theta ,k,m)}_{k}(x+B_dW^{d,(\theta ,k,m)}_{R^{(\theta ,k,m)}}) \nonumber \\&- U^{d,(\theta ,k,-m)}_{k-1}(x+B_dW^{d,(\theta ,k,m)}_{R^{(\theta ,k,m)}}) \Big ] \nonumber \\&- \Big [ f_d\!\left( x+B_dW^{d,(\theta ,k,m)}_{R^{(\theta ,k,m)}}, U^{d,(\theta ,k,m)}_{k}(x+B_dW^{d,(\theta ,k,m)}_{R^{(\theta ,k,m)}}) \right) \nonumber \\&- f_d\!\left( x+B_dW^{d,(\theta ,k,m)}_{R^{(\theta ,k,m)}}, U^{d,(\theta ,k,-m)}_{k-1}(x+B_dW^{d,(\theta ,k,m)}_{R^{(\theta ,k,m)}}) \right) \Big ] \bigg ) \Bigg ], \end{aligned}$$

and let $\mathfrak {C}_{d,n} \in \mathbb {R}$, $d,n\in \mathbb {N}_0$, satisfy for all $d,n \in \mathbb {N}_0$ that $\mathfrak {C}_{d,n} \leqslant (d+1) M^n + \sum _{k=1}^{n-1} M^{n-k} ( d + 1 + \mathfrak {C}_{d,k} + \mathfrak {C}_{d,k-1} )$. Then there exist $c \in \mathbb {R}$ and $\mathfrak {N} :(0,1] \times \mathbb {N}\rightarrow (\mathbb {Z}\cap [\mathfrak {n},\infty ))$ such that for all $\varepsilon \in (0,1]$, $d \in \mathbb {N}$ it holds that $\mathfrak {C}_{d,\mathfrak {N}_{\varepsilon ,d}} \leqslant c d^{1 + \alpha ( s + p \max \{q,2r+1\} )} {\varepsilon }^{-\alpha }$ and190

#### Proof of Theorem 3.16

Throughout this proof let $\lceil \cdot \rceil :\mathbb {R}\rightarrow \mathbb {Z}$ satisfy for all $x \in \mathbb {R}$ that $\lceil x \rceil = \min ( \mathbb {Z}\cap [x,\infty ) )$, let $c = \frac{4\kappa ((\lceil p\rceil )!)}{(\sqrt{\lambda }-\sqrt{L})^2} \max \left\{ 1,\left[ \frac{8|\kappa |^2}{\lambda -L}\right] ^{p} \right\} \exp ( 1 + \kappa ),$ let , and let $\eta _d \in (0,\infty )$, $d \in \mathbb {N}$, satisfy for all $d \in \mathbb {N}$ that $\eta _d = \sup \{ t \in [0,\infty ) :(8|\kappa |^2 d^{2r+1} t \leqslant \lambda - L~\text {and}~ t d^q \leqslant 1) \}$. Observe that the fact that for all $d \in \mathbb {N}$ it holds that $\eta _d \leqslant 1$, the fact that for all $d \in \mathbb {N}$, $x \in \mathbb {R}^d$ it holds that $\left\| B_d x \right\| \leqslant \kappa d^r \left\| x \right\|$, and the fact that for all $d \in \mathbb {N}$ it holds that $8 \eta _d |\kappa |^2 d^{2r+1} \leqslant \lambda - L$ ensure that for all $d \in \mathbb {N}$ it holds that

191 $$\begin{aligned} \begin{aligned} |\eta _d|^2 \left[ \sup _{x\in \mathbb {R}^d{\setminus }\{0\}} \left( \frac{ \left\| B_dx \right\| }{ \left\| x \right\| }\right) \right] ^2&\leqslant d \eta _d \left[ \sup _{x\in \mathbb {R}^d{\setminus }\{0\}} \left( \frac{ \left\| B_dx \right\| }{ \left\| x \right\| }\right) \right] ^2 \\  &\leqslant \eta _d |\kappa |^2 d^{2r+1} \leqslant \frac{\lambda - L}{8}. \end{aligned} \end{aligned}$$

This implies for all $d \in \mathbb {N}$ that

192 $$\begin{aligned} \lambda - L - 2(|\eta _d|^2+d\eta _d) \left[ \sup _{x\in \mathbb {R}^d{\setminus }\{0\}} \left( \frac{ \left\| B_d x \right\| }{ \left\| x \right\| }\right) \right] ^2 \geqslant \frac{\lambda - L}{2}. \end{aligned}$$

Next note that the assumption that for all $d \in \mathbb {N}$, $\varepsilon \in (0,\infty )$ it holds that $\sup _{ x \in \mathbb {R}^d } [ |u_d(x)| \exp (-\varepsilon \left\| x \right\| )] < \infty$ proves that for all $d \in \mathbb {N}$, $\varepsilon \in (0,\infty )$ it holds that

193 $$\begin{aligned} \begin{aligned}&\sup _{x=(x_1,\ldots ,x_d)\in \mathbb {R}^d} \left[ \frac{|u_d(x)|}{\exp (\varepsilon \sum _{i=1}^d |x_i| )} \right] \leqslant \sup _{x\in \mathbb {R}^d} \left[ \frac{|u_d(x)|}{\exp (\varepsilon \Vert x \Vert )} \right] < \infty . \end{aligned} \end{aligned}$$

Corollary 3.13 therefore ensures for all $d \in \mathbb {N}$, $n \in \mathbb {N}_0$, $x \in \mathbb {R}^d$ that194The fact that for all $x \in (0,\infty )$ it holds that $x^p \leqslant ((\lceil p \rceil )!)\exp (x)$ and the fact that for all $d \in \mathbb {N}$ it holds that $\eta _d d^q \leqslant 1$ hence imply that for all $d \in \mathbb {N}$, $n \in \mathbb {N}_0$ it holds that195Moreover, observe that Lemma 3.14 (with $\alpha \leftarrow d+1$, $\beta \leftarrow d+1$, $M \leftarrow M$, $(C_n)_{ n \in \mathbb {N}_0 } \leftarrow (\max \{\mathfrak {C}_{d,n},0\})_{ n \in \mathbb {N}_0 }$ for $d \in \mathbb {N}$ in the notation of Lemma 3.14) ensures for all $d,n \in \mathbb {N}$ that $\mathfrak {C}_{d,n} \leqslant 3d(3\,M)^n$. Combining this with the fact that for all $d \in \mathbb {N}$ it holds that $\mathfrak {C}_{d,0} \leqslant d+1 \leqslant 3d$ implies that for all $d \in \mathbb {N}$, $n \in \mathbb {N}_0$ it holds that $\mathfrak {C}_{d,n} \leqslant 3d(3M)^n$. Next let $N :(0,1] \times \mathbb {N}\rightarrow \mathbb {N}_0$ satisfy for all $d \in \mathbb {N}$, $\varepsilon \in (0,1]$ that196Lemma 3.15 (with , $\beta \leftarrow 3\,M$, $\kappa _1 \leftarrow c d^{s+p\max \{q,2r+1\}}$, $\kappa _2 \leftarrow 3d$, $m \leftarrow \lceil \mathfrak {n}\rceil$, , $(c_n)_{n\in \mathbb {Z}\cap [ \mathfrak {n},\infty )} \leftarrow (\max \{ \mathfrak {C}_{d,n}, 0\} )_{n\in \mathbb {Z}\cap [ \mathfrak {n},\infty )}$ for $d \in \mathbb {N}$ in the notation of Lemma 3.15), the fact that for all $d \in \mathbb {N}$, $n \in \mathbb {N}_0$ it holds that $\mathfrak {C}_{d,n} \leqslant 3d(3\,M)^n$, and (195) therefore imply that for all $d \in \mathbb {N}$, $\varepsilon \in (0,1]$ it holds that  and

197 $$\begin{aligned} \begin{aligned} \mathfrak {C}_{d,N_{\varepsilon , d}}&\leqslant 3 d \max \left\{ (3M)^{\lceil \mathfrak {n} \rceil }, 3M (c d^{ s + p \max \{ q, 2r+1 \} })^{\alpha } \right\} \left[ \frac{1}{\varepsilon } \right] ^{\alpha } \\&\leqslant \max \left\{ 3 (3M)^{\lceil \mathfrak {n} \rceil }, 9M c^{\alpha } \right\} d^{ 1 + ( s + p \max \{ q, 2r+1 \} ) \,\alpha } \left[ \frac{1}{\varepsilon } \right] ^{\alpha }. \end{aligned} \end{aligned}$$

This establishes (190). The proof of Theorem 3.16 is thus completed. $\square$

#### Corollary 3.17

Let $c,L \in [0,\infty )$, $\lambda \in (L,\infty )$, $M \in \mathbb {N}\cap ( (\sqrt{\lambda }+\sqrt{L})^2(\sqrt{\lambda }-\sqrt{L})^{-2}, \infty )$, $\Theta = \cup _{n\in \mathbb {N}} \mathbb {Z}^n$, let $u_d \in C(\mathbb {R}^d,\mathbb {R})$, $d \in \mathbb {N}$, let $B_d \in \mathbb {R}^{d\times d}$, $d \in \mathbb {N}$, let $f_d \in C(\mathbb {R}^d\times \mathbb {R},\mathbb {R})$, $d \in \mathbb {N}$, assume for every $d \in \mathbb {N}$ that $u_d$ is a viscosity solution of

198 $$\begin{aligned} \tfrac{1}{2}{\text {Trace}}(B_d(B_d)^{*}({\text {Hess}} u_d)(x)) = f_d(x,u_d(x)) \end{aligned}$$

for $x \in \mathbb {R}^d$, let $( \Omega , \mathcal {F}, \mathbb {P})$ be a probability space, let $W^{d,\theta } :[0,\infty ) \times \Omega \rightarrow \mathbb {R}^d$, $\theta \in \Theta$, $d \in \mathbb {N}$, be i.i.d. standard Brownian motions, let $R^{\theta } :\Omega \rightarrow [0,\infty )$, $\theta \in \Theta$, be i.i.d. random variables, assume that $(R^{\theta })_{\theta \in \Theta }$ and $(W^{d,\theta })_{(d,\theta )\in \mathbb {N}\times \Theta }$ are independent, let $\left\| \cdot \right\| :(\cup _{d\in \mathbb {N}} \mathbb {R}^d) \rightarrow [0,\infty )$ satisfy for all $d \in \mathbb {N}$, $x = (x_1,\ldots ,x_d) \in \mathbb {R}^d$ that , assume for all $d \in \mathbb {N}$, $x \in \mathbb {R}^d$, $v,w \in \mathbb {R}$, $\varepsilon \in (0,\infty )$ that $\left\| B_dx \right\| \leqslant c d^{c} \Vert x \Vert$, $| f_d(x,v) - f_d(x,w) - \lambda ( v - w ) | \leqslant L | v-w |$, $|f_d(x,0)| \leqslant c d^{c} (1+ \left\| x \right\| ^c)$, $\sup _{y\in \mathbb {R}^d} [|u_d(y)|\exp (-\varepsilon \Vert y \Vert )] < \infty$, and

199 $$\begin{aligned} \textstyle \mathbb {P}(R^0 \geqslant \varepsilon ) = e^{-\lambda \varepsilon }, \end{aligned}$$

let $U^{d,\theta }_{n} = (U^{d,\theta }_{n}(x))_{x\in \mathbb {R}^d}:\mathbb {R}^d\times \Omega \rightarrow \mathbb {R}$, $\theta \in \Theta$, $d,n\in \mathbb {N}_0$, satisfy for all $d,n \in \mathbb {N}$, $\theta \in \Theta$, $x \in \mathbb {R}^d$ that $U^{d,\theta }_{0}(x) = 0$ and

200 $$\begin{aligned} \begin{aligned} U^{d,\theta }_{n}(x)&= \frac{-1}{\lambda M^n} \left[ \sum _{m=1}^{M^n} f_d(x+B_dW^{d,(\theta ,0,m)}_{R^{(\theta ,0,m)}},0) \right] \\&+ \sum _{k=1}^{n-1} \frac{1}{\lambda M^{n-k}} \Bigg [ \sum _{m=1}^ {M^{n-k}} \bigg ( \lambda \Big [ U^{d,(\theta ,k,m)}_{k}(x+B_dW^{d,(\theta ,k,m)}_{R^{(\theta ,k,m)}}) \\  &- U^{d,(\theta ,k,-m)}_{k-1}(x+B_dW^{d,(\theta ,k,m)}_{R^{(\theta ,k,m)}}) \Big ] \\  &- \Big [ f_d\!\left( x+B_dW^{d,(\theta ,k,m)}_{R^{(\theta ,k,m)}}, U^{d,(\theta ,k,m)}_{k}(x+B_dW^{d,(\theta ,k,m)}_{R^{(\theta ,k,m)}}) \right) \\&- f_d\!\left( x+B_dW^{d,(\theta ,k,m)}_{R^{(\theta ,k,m)}}, U^{d,(\theta ,k,-m)}_{k-1}(x+B_dW^{d,(\theta ,k,m)}_{R^{(\theta ,k,m)}}) \right) \Big ] \bigg ) \Bigg ], \end{aligned} \end{aligned}$$

and let $\mathfrak {C}_{d,n} \in \mathbb {R}$, $d,n\in \mathbb {N}_0$, satisfy for all $d,n \in \mathbb {N}_0$ that $\mathfrak {C}_{d,n} \leqslant (d+1) M^n + \sum _{k=1}^{n-1} M^{n-k} ( d + 1 + \mathfrak {C}_{d,k} + \mathfrak {C}_{d,k-1} )$. Then there exist $\kappa \in \mathbb {R}$ and $\mathfrak {N} :(0,1] \times \mathbb {N}\rightarrow \mathbb {N}$ such that for all $\varepsilon \in (0,1]$, $d \in \mathbb {N}$ it holds that201

#### Proof of Corollary 3.17

Corollary 3.17 is an immediate consequence of Theorem 3.16. A detailed proof can be found, e.g., in the extended preprint version [7, Corollary 3.17] of this article. $\square$

## Data Availability

No data were created or analyzed in this article.
